# Tooth Removal in the Leopard Gecko and the *de novo* Formation of Replacement Teeth

**DOI:** 10.3389/fphys.2021.576816

**Published:** 2021-05-04

**Authors:** Kirstin S. Brink, Joaquín Ignacio Henríquez, Theresa M. Grieco, Jesus Rodolfo Martin del Campo, Katherine Fu, Joy M. Richman

**Affiliations:** Department of Oral Health Sciences, Life Sciences Institute, University of British Columbia, Vancouver, BC, Canada

**Keywords:** reptile, pulse-chase, label-retaining cell, dentition, polyphyodont, successional teeth, adult tissue stem cells, dental epithelium

## Abstract

Many reptiles are able to continuously replace their teeth through life, an ability attributed to the existence of epithelial stem cells. Tooth replacement occurs in a spatially and temporally regulated manner, suggesting the involvement of diffusible factors, potentially over long distances. Here, we locally disrupted tooth replacement in the leopard gecko (*Eublepharis macularius*) and followed the recovery of the dentition. We looked at the effects on local patterning and functionally tested whether putative epithelial stem cells can give rise to multiple cell types in the enamel organs of new teeth. Second generation teeth with enamel and dentine were removed from adult geckos. The dental lamina was either left intact or disrupted in order to interfere with local patterning cues. The dentition began to reform by 1 month and was nearly recovered by 2–3 months as shown in μCT scans and eruption of teeth labeled with fluorescent markers. Microscopic analysis showed that the dental lamina was fully healed by 1 month. The deepest parts of the dental lamina retained odontogenic identity as shown by PITX2 staining. A pulse-chase was carried out to label cells that were stimulated to enter the cell cycle and then would carry BrdU forward into subsequent tooth generations. Initially we labeled 70–78% of PCNA cells with BrdU. After a 1-month chase, the percentage of BrdU + PCNA labeled cells in the dental lamina had dropped to 10%, consistent with the dilution of the label. There was also a population of single, BrdU-labeled cells present up to 2 months post surgery. These BrdU-labeled cells were almost entirely located in the dental lamina and were the likely progenitor/stem cells because they had not entered the cell cycle. In contrast fragmented BrdU was seen in the PCNA-positive, proliferating enamel organs. Homeostasis and recovery of the gecko dentition was therefore mediated by a stable population of epithelial stem cells in the dental lamina.

## Introduction

Polyphyodonty, or life-long tooth replacement, is a developmental process shared by most non-mammalian vertebrates. In polyphyodont reptiles, it has been well documented that replacement occurs in waves that pass from the back to the front of the mouth in alternating tooth positions (Edmund, 1960, 1962; Cooper, 1966; Kline and Cullum, 1984; Kline and Cullum, 1985; Fastnacht, 2008; Grieco and Richman, 2018; Hanai and Tsuihiji, 2019). One key component of the expression of these jaw-level replacement waves is that each tooth position cycles on a temporally delayed schedule from adjacent positions. An examination of tooth replacement in young leopard geckos demonstrated that patterning is initiated *in ovo* and continuous in post-hatching animals, pointing to some conserved mechanisms at the patterning level in adults (Grieco and Richman, 2018). However, the mechanisms behind this cyclical tooth replacement are still not well understood.

Many studies have investigated potential mechanisms for the establishment of this alternating wave replacement pattern in polyphyodont animals. In fish, there is strong evidence for signals that originate with an initiator tooth at the front of the jaw and pattern the tooth row or rows (Huysseune et al., 2012; Gibert et al., 2019; Sadier et al., 2020). This mechanism has also been suggested for reptiles, however, it has not been tested (Edmund, 1969). Other potential mechanisms proposed for reptiles include a reaction-diffusion model or ‘zone of inhibition’ model that creates spacing between teeth that change through growth, creating an emergent alternating replacement pattern (Osborn, 1970, 1971; Westergaard and Ferguson, 1987; Murray and Kulesa, 1996; Fraser et al., 2008). These models have all been proposed based on examination of tooth initiation in embryos, and so it is still not known how patterns of tooth cycling are maintained in adults.

Tooth replacement in polyphyodont reptiles can be partially explained by the persistence of dental epithelium throughout life (Handrigan et al., 2010; Wu et al., 2013; Salomies et al., 2019; Brink et al., 2020). In reptiles the dental lamina may either be continuous around the jaws as in lizards and snakes, or be focal islands of epithelial cells next to the functional tooth as in crocodilians. In contrast, in most mammals, the dental epithelium undergoes apoptosis prenatally (Buchtova et al., 2012) and only a few cells posterior to the terminal tooth in the arch or in the periodontal ligament persist after birth. In the leopard gecko, tooth development begins at the most aboral end (furthest away from the oral cavity) or free-end of the dental lamina. The early bud passes through typical stages of tooth development (bud, cap, bell, histodifferentiation, eruption) ultimately emerging into the oral cavity. The dental lamina connects the functional tooth to the underlying replacement teeth at each tooth position. The dental lamina continues to grow on the lingual side of the youngest tooth to form the successional lamina. Each new tooth buds from the successional lamina and the cycle begins again. The ectomesenchyme condenses around the bud, and both tissues interact, to undergo morphogenesis and histodifferentiation (Handrigan et al., 2010; Handrigan and Richman, 2011).

The dental lamina is the likely source of epithelial stem cells in polyphyodont reptiles (Handrigan et al., 2010; Wu et al., 2013). Previous work in leopard geckos found that the majority of label-retaining cells (putative stem cells) were located on the lingual side of the dental lamina, approximately half-way between the successional lamina and the oral cavity (Handrigan et al., 2010). Label-retaining cells were not observed streaming into the generational teeth but instead appeared to be quiescent and isolated from other BrdU-positive cells. However, putative stem cells may have other niches close to the oral epithelium, as shown in the bearded dragon (Salomies et al., 2019). Label-retaining cells were present in low numbers in the successional lamina of the bearded dragon (Salomies et al., 2019) and iguana (Brink et al., 2020) but were only seen in the dental lamina bulge (successional lamina) of the American alligator in the pre-initiation stage of tooth development (Wu et al., 2013). The differences in proximity to the successional teeth and scarcity of these label-retaining cells means that there are still important questions to address regarding the fates (or roles) of these putative stem cells in development of the next generation of teeth.

Previously, there were studies that attempted to stimulate tooth replacement by removing functional teeth. In alligators, an increase in proliferation was observed in the dental lamina bulge (Wu et al., 2013). This response in the deepest part of the dental lamina could have led to the early differentiation of the successional lamina, however long term follow up was not included in this study (Wu et al., 2013). In the green iguana, teeth were also initiated earlier than expected after extraction of functional teeth (Brink et al., 2020). However, after early initiation, the timing of the tooth cycle was not affected. Because these iguana studies focused on individual functional teeth at different positions around the mouth, they could not provide information on the relationships between adjacent teeth or mechanisms contributing to jaw-wide patterning. Therefore, it is still not understood how disruptions to odontogenic tissues and developing replacement teeth affect the timing and patterning of replacement in the context of the most prevalent models for tooth replacement.

In this study, we aimed to track the effect of the removal of second generation teeth and damage to the dental lamina on the development of subsequent tooth generations using the leopard gecko, which has a known tooth shedding cycle length (Grieco and Richman, 2018) and populations of label-retaining cells in the dental lamina (Handrigan et al., 2010). We ask whether or not the removal of the second generation teeth will affect the patterning of neighboring teeth, whether or not the purported stem cells from the dental lamina (distant from the location of the next tooth bud) give rise to the next tooth generation, and whether or not the dental lamina cells can give rise to ameloblasts, stellate reticulum, and outer enamel epithelium of the enamel organs. We hypothesized that teeth would regenerate after healing of the dental lamina, but that patterning would be disrupted due to removal of signaling between adjacent teeth within the surgical area. We also hypothesized that epithelial stem cells from the dental lamina would contribute to all layers of the enamel organ in the newly formed teeth. Our results show that patterning is not greatly affected following removal of second generation teeth, and that the process of tooth regeneration is robust in the gecko since non-proliferative, dental epithelial progenitor cells are maintained in the dental lamina. In addition, cells derived from the original labeled dental lamina contributed to all layers of the enamel organ.

## Materials and Methods

### Survival Surgeries on Adult Geckos

All animal procedures were approved by the UBC Animal Care Committee (ethics protocol A15-0242). The colony of leopard geckos is housed in a dedicated facility with 12 h light-dark cycles and heated cages. Animals were raised from hatching or purchased from retail stores. Eighteen geckos (15 female and 3 male) ranging in age from 2 months to 1-year post-hatching and weighing 13–75 g were subjected to oral surgeries. Some individuals were used for experiments at multiple time points in the maxillae and mandibles (Supplementary Table 1). Survival surgeries were carried out in an animal surgical suite with an isofluorane vaporizer, O_2_ mixer, scavenger system for the gas and fluorescence stereomicroscope (MZFLIII).

Geckos were given 0.2 mg/kg meloxicam orally and 15 mg/kg calcein (Sigma-Aldrich, cat no. C0875), via intraperitoneal injection 24 h before the surgery. The fluorescent calcein label is rapidly incorporated into mineralizing enamel, dentine and bone. Animals were induced with a mixture of 5% isoflurane/O_2_ gas under continuous flow and intramuscular injections of 20 mg/kg alfaxalone were delivered bilaterally into the epaxial muscles. Local anesthetic was administered into the palate or mandible prior to surgery via injection (0.5% lidocaine, 7 mg/kg maximum dose).

**FIGURE 1:**
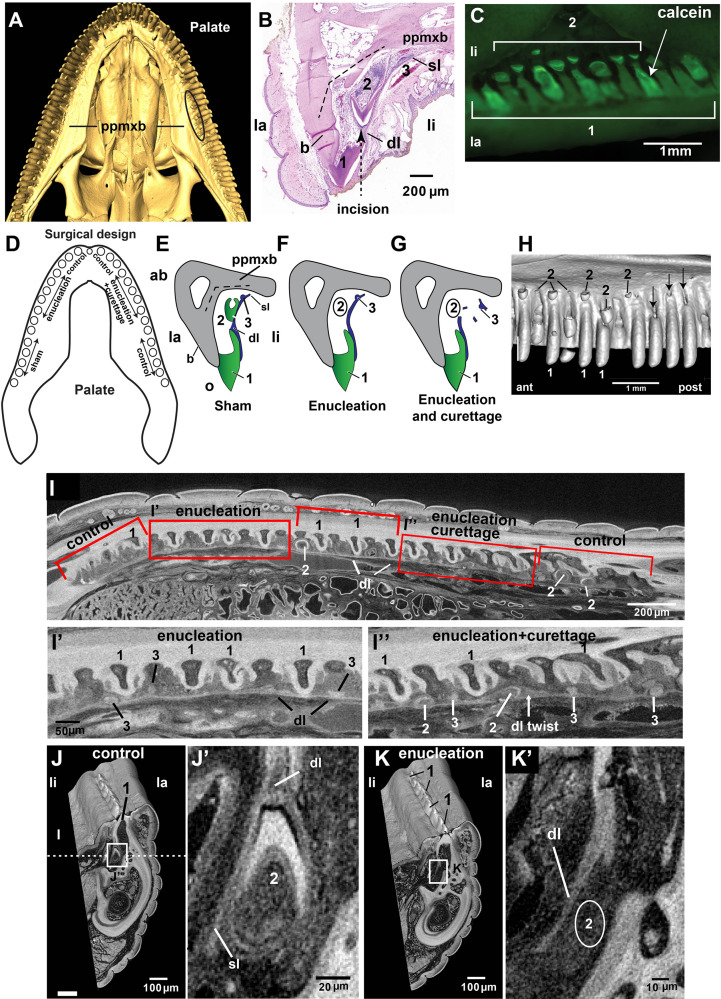
The polyphyodont leopard gecko model. **(A)** μCT scan of leopard gecko maxilla showing replacement teeth close to the angle between the dental and palatal processes of the maxillary bone (oval outline). The mineralized replacement teeth are found around the arch at different stages of development. **(B)** Hematoxylin and Eosin-stained section of the leopard gecko maxilla, transverse plane. The mineralized first generation or functional tooth is connected to the second generation and third generation teeth by a dental lamina. The incision made lingual to the 1st generation tooth exposes the second generation tooth (dashed line with arrowhead). The projection of the palatine process of the maxillary bone is shown by the angled dashed line. This is anatomical reference for where to expect new teeth to form. **(C)** Calcein-labeled teeth at the time of surgery. The mucosa has been incised and retracted to show the labeled, unerupted second generation teeth. **(D)** Experimental design for tooth removal surgeries in the maxilla, which involves multiple sites of 10 teeth each. Third generation teeth form close to the palatine process of the maxillary bone. **(E)** Schematic to show the sham surgical control, with the oral mucosa cut and retracted but teeth and soft tissues not disturbed. **(F)** Enucleation involves removal of the second generation tooth (2 inside a circle) while leaving the dental lamina and third generation teeth in place. **(G)** enucleation with curettage, comprising removal of second generation teeth (2 inside a circle) and curettage of dental tissues, damaging the dental and successional laminae. **(H)** Lingual view of a region of a μCT scan showing the specificity of the surgery. A group of teeth have been removed posteriorly while the anterior second generation teeth remain undisturbed. **(I–K’)** Contrast-enhanced μCT scan of a 1-week post-surgery mandible. Several regions were treated in one quadrant to maximize information obtained from this specimen. **(I’)** Enucleation surgical area in frontal view showing third generation replacement teeth present and a straight dental lamina. **(I”)** Enucleation with curettage surgical area in frontal view showing the missing and displaced replacement teeth as well as a thicker dental lamina that may be twisted. **(J,J’)** Transverse slice through the control area showing a second generation tooth and dental lamina. Dashed line indicates plane of section in **(I–I”)**. **(K,K’)** Transverse slice through the enucleated area showing the absent second generation tooth plus remaining dental lamina. Key: 1- functional, 1st generation tooth; 2- second generation replacement tooth; 3- third generation replacement tooth; ab, aboral; ant, anterior; b, bone; dl, dental lamina; dl twist, twisted dental lamina; la, labial; li, lingual; o, oral; post, posterior; ppmxb, palatine process of maxillary bone; sl, successional lamina.

Quadrants of the mouth were divided into different treatment regions ranging from 10 to 20 teeth: (1) enucleation (surgical removal) of developing second generation teeth containing mineralized tissue while leaving the non-mineralized, third generation bud stage teeth, (2) enucleation of second generation teeth plus curetting the surrounding tissues, thereby removing and/or displacing those tissues and non-mineralized third generation bud stage teeth, and (3) sham surgical areas, where the lingual mucosa was cut next to the teeth and retracted lingually with minimal damage to adjacent tissues (Figures 1B–H). Adjacent control, non-manipulated regions were also studied to assess the normal patterns of patterning and proliferation.

**FIGURE 2:**
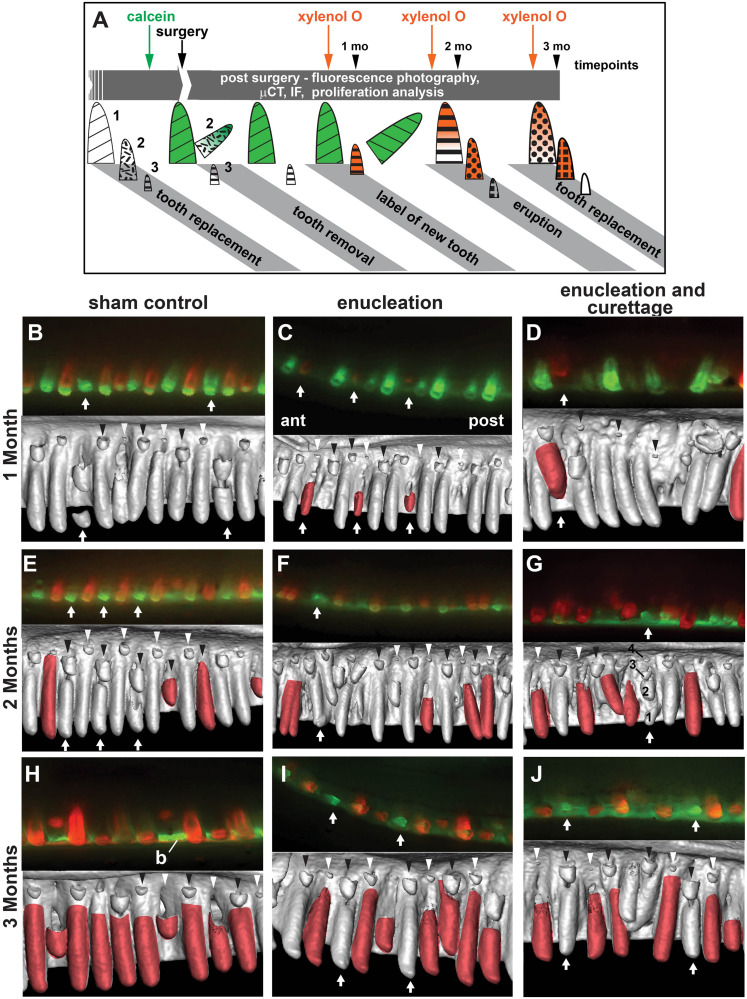
Tooth regeneration dynamics. **(A)** Schematic of the experiment starting on the left. Calcein was administered once, 24 h before second generation tooth removal. Functional teeth and second generation teeth are labeled green. Third generation teeth are not mineralized and do not take up the label. Xylenol orange was administered 1 week prior to the 1, 2, and 3 months timepoints (euthanization). Orange teeth are mineralized after the surgery. Fill patterns correspond to specific tooth generations. **(B–J)** Top half of each panel are fluorescent images taken after fixation with all soft tissues present. Signal is partially obscured by the mucosa. The fluorescent orange teeth were matched up to the equivalent teeth in the μCT images (bottom half) and only teeth with exclusive orange label were false colored. Black and white arrowheads indicate replacement waves or incipient replacement waves in alternating tooth positions. **(B)** Sham control – Most teeth are double labeled with green tips and an orange base with the exception of two teeth that are green only and are about to be replaced with orange teeth (arrows). **(C)** Enucleation – three teeth are completely orange and are newly erupting (arrows). **(D)** Enucleation with curettage – A newly-erupting, orange tooth is visible (arrow). **(E)** At 2 months post-surgery in the sham control, many teeth are double labeled and several erupted teeth are exclusively orange. The green teeth will soon be replaced (arrows). **(F,G)** 2 months following enucleation or enucleation and curettage, double-labeled teeth are present, exclusively orange teeth are present, and occasional green teeth are still present (arrows). The μCT scans show some third generation teeth are out-of-place. In **(G)**, one tooth position has 3 successional teeth (arrow). **(H)** At 3 months post-surgery in the sham control, all teeth are orange, indicating that they have all been replaced since the surgery. The visible green is in the underlying jawbone. **(I,J)** After enucleation or enucleation and curettage some of the original green teeth have been retained in the mouth longer than expected when compared to controls (arrows). The μCT scans show third generation teeth are initiating in a somewhat normal alternating pattern. Key: ant, anterior; b, bone; post, posterior.

Iridectomy scissors were inserted into the marginal mucosa lingual to the functional teeth (Figure 1B). The mucosa was retracted with a blunt instrument (Dycal applicator, dental instrument) to view developing second generation teeth containing calcein (GFP filter set; Figure 1C). Third generation teeth were not visible because they were not mineralized. Several sites were treated in one animal (Figure 1D). In the experimental regions, mineralized, unerupted teeth were removed using #5 fine forceps with (enucleation + curettage) or without (enucleation) disruption of the dental epithelium (Figures 1E–G). For curettage, a spoon excavator (dental instrument) was moved anterior-posteriorly under the mucosa to tear and shift the position of the dental lamina. Affi-Gel blue beads (BioRad) were implanted at each end of the incision to mark the surgical area in subsequent histological analysis. Photographs were taken using a fluorescent and brightfield stereo microscope before and after surgery. One week before euthanization, six animals were administered 90 mg/kg of xylenol orange (Sigma-Aldrich, cat. No. 398187) through intraperitoneal injection to label teeth that mineralized after surgery (Figure 2A).

### BrdU Pulse-Chase Experiment

Animals were administered 50 mg/kg Bromo-deoxyuridine (BrdU, Cat no. B5002, Sigma-Aldrich) intraperitoneally once a day for 4 consecutive days, beginning 3 days after surgery. Animals were euthanized as described at time zero (right after the 4-day pulse or 1 week post surgery), 3 weeks post BrdU (1 month post surgery), and 7 weeks post BrdU (8 weeks post-surgery).

### μCT Scanning

Following euthanasia with isoflurane, gecko heads were fixed in 4% paraformaldehyde for at least 24 h at 4°C, rinsed in saline, embedded in 2% agarose, and dehydrated into 70% ethanol. Skulls were μCT scanned in the Centre for High Throughput Phenogenomics (CHTP) at the University of British Columbia on a Scanco Medical μCT100 at a resolution of 17 μm. A mandible from 1 animal was treated in a 0.7% Phosphotungstic Acid (PTA)-Methanol solution for 7 days in order to increase the radio-contrast of its soft tissues, following Metscher (2009). The mandible was μCT scanned at the Central European Institute of Technology (CEITEC) at Brno University of Technology on a GE phoenix v| tome| x m at a resolution of 4 μm. PTA staining is incompatible with histological or molecular analyses. All maxillary specimens were reserved for molecular studies.

### Histology and Immunofluorescence Staining

After standard μCT scanning, the gecko maxilla and mandible pieces were divided into segments using landmarks recorded during surgery. Pieces were decalcified in 14% EDTA for at least 3 months at room temperature on a shaker prior to processing into paraffin wax. To determine the areas where surgery had been carried out, test slides were made. Every 10th section throughout the block was stained with Hematoxylin and Eosin. Selected 7 μm sections from multiple blocks were placed on slides so that the staining conditions were equivalent for a variety of treatments. Multiple replicates of teeth were put across several slides, further validating the results (Supplementary Table 2).

Sections were deparaffinized and rehydrated to water. Antigen retrieval for all immunostaining was performed using Diva Decloaker (Biocare #DV2004MX). For combination BrdU and PCNA staining, sections were pretreated with 0.1% Triton-X in PBS for 10 min and then blocked in 10% goat serum, 0.1% Triton-X in PBS for 30 min. Anti-BrdU (G3G4 clone, Developmental Studies Hybridoma Bank, mouse 1:20) and anti-PCNA (Proliferating Cell Nuclear Antigen, rabbit polyclonal, Abcam, #18197, 1:1000) were mixed together in blocking solution and incubated overnight at 4°C.

For PITX2, Keratin, and SOX2 staining sections were blocked in 5% bovine serum, 0.1% Tween-20 in TBS for 1 h at room temperature. Tissues were incubated overnight at 4°C in primary antibodies diluted in blocking serum (sheep polyclonal anti-PITX2, R&D Systems, #AF7388, 1:500; anti-SOX2, rabbit polyclonal, Abcam # 97959, 1:1000; rabbit anti Pan-Keratin, Dako, cat no. Z062201-2, 1:500).

Secondary antibodies were applied for 1 h at room temperature (all diluted 1:250, Life Technologies, Alexa Fluor 647 donkey anti-sheep, #A-21488; Alexa Fluor 488 goat anti-mouse, #A11029; Cy5 goat anti-rabbit, #A10523). Nuclei were counterstained in Hoescht 33258 (Sigma, 10 μg/ml) for 30 min. Slides were mounted with Prolong Gold (Invitrogen #P36934). Fluorescence imaging was conducted using a 20X objective on a Panoramic MIDI II slide scanner with 488, Cy5, and DAPI filter sets (3D Histech Ltd., Hungary). Images were captured with CaseViewer software v.2.4.0.119028 (3D Histech Ltd., Hungary).

### Proliferation Analysis

We used CaseViewer software to count BrdU-, PCNA- and Hoechst-stained nuclei in the dental laminae. By turning on and off the channels, dual-labeled nuclei could be confirmed. The total number of green, red or dual-labeled cells was divided by the number of Hoechst-stained cells in that specimen to give the percent label for that dental lamina. Care was taken to space apart the paraffin sections by at least 250 microns (the width of a functional tooth) so that teeth from different families were being analyzed. These teeth rather than the animals were considered individual biological replicates. Teeth for each of the experimental conditions (enucleated, enucleation + curettage, sham or control) were combined from three animals (Supplementary Table 2). We compared percent label at two time points (Time zero and 1 month) as well as between the four treatments using two-way ANOVA (Supplementary Tables 3A–D). Statistical analyses were carried out using GraphPad Prism 9.0.2.

For animals euthanized at 2 months, few labeled cells were present, therefore we did not calculate percentages (Supplementary Table 4). Instead, we determined the raw number of labeled cells in different regions of the enamel organ (inner enamel epithelium, outer enamel epithelium, and stellate reticulum) and the dental lamina of bud or cap stage teeth (Supplementary Table 4).

### TUNEL Assay

Apoptosis was assessed by TUNEL analysis using the ApopTag Apoptosis Kit (Millipore #S7111) and was detected using fluorescein-tagged anti-digoxigenin antibody as described previously (Hosseini-Farahabadi et al., 2013). A qualitative assessment of presence of TUNEL-positive cells was carried out.

## Results

Our goal in this study was to challenge the adult gecko dentition by purposely disrupting tooth replacement, thus initiating a healing response. We left functional teeth in place and instead removed or enucleated the unerupted, partly mineralized (second generation) replacement teeth in select regions of the jaw of the leopard gecko (Figure 1). The aim was to leave the dental lamina behind plus or minus immature third generation teeth (non-mineralized bud or early cap stage). In more advanced cap stage teeth, the successional lamina may be present. The aim was to test the regenerative capacity of the dental epithelium inside the jaws.

The adult leopard gecko has approximately 40 functional teeth in each quadrant (Figure 1A) and in the maxilla each functional tooth is connected to two successional teeth via a dental epithelial lamina (Figure 1B). The second generation, mineralized, unerupted teeth are nestled at the inflection between the dental and palatine processes of the maxillary bone (Figure 1A) and are at various stages of crown formation (Figure 1A). In addition, each family contains a tooth in bud or early cap stage prior to cytodifferentiation (third generation) that is visible only in histology (Figure 1B). The successional lamina is connected to the outer enamel epithelium of the second generation tooth and continues to initiate the third generation of teeth (Figure 1B). For direct visualization of second generation teeth during surgery, we injected calcein which was rapidly taken up in the dentine and enamel caps (Figure 1C). The three treatments – sham, enucleation and enucleation, and curettage (Figures 1D–G) – started with an incision lingual to the functional tooth (Figure 1B). This incision gave direct access to second generation teeth (Figure 1C). We confirmed that teeth were removed using conventional μCT scans for all specimens that were subsequently used for histology (Figure 1H). One mandibular quadrant was treated with the two surgeries, fixed after 1 week, then stained with phosphotungstic acid prior to scanning at high resolution (Figures 1I–K’). Mineralized second generation replacement teeth are still present in the control areas (Figures 1I,J,J’). However, in the enucleated areas, second generation replacement teeth were successfully removed (Figures 1K,K’), and soft tissues with third generation teeth are still present (Figures 1I–I”). Buds are evenly spaced in the enucleated area, while in the enucleated area with curettage, some buds are bunched together at the edges of the treatment site and the dental lamina is thicker and therefore likely to be twisted (Figure 1I”).

### Recovery of Normal Patterns of Tooth Replacement by 3 Months

Next, we followed the recovery of the dentition. Animals were injected with calcein 24 h before surgery and were injected with xylenol orange 1 week before euthanization to label teeth that were formed *de novo* post-surgery (Figure 2A). After 1 month, the control areas contained mainly teeth with a double label (green tip and orange base). The dual label indicated that these teeth were present prior to the surgery (green) and had added new dentine since the surgery (orange). In the subsequent μCT scan of the matched region, second generation teeth are visible in an alternating wave pattern of small and large teeth, increasing in size toward the back of the jaw (Figure 2B, black and white arrowheads). When compared to the μCT scan, the green teeth are about to be replaced and were not actively mineralizing (Figure 2B, arrows). In both treated areas, new teeth labeled with xylenol orange were superficial enough to make them visible through the mucosa (Figures 2C,D, white arrows). Enucleation and curettage interfered with the patterning of *de novo* tooth formation (Figure 2D, black arrowheads) more severely than enucleation alone (Figure 2C, black and white arrowheads). In addition, teeth are crowded together and oriented abnormally, suggesting that tooth initiation and development were occurring in displaced tissues (Figure 2D).

**FIGURE 3:**
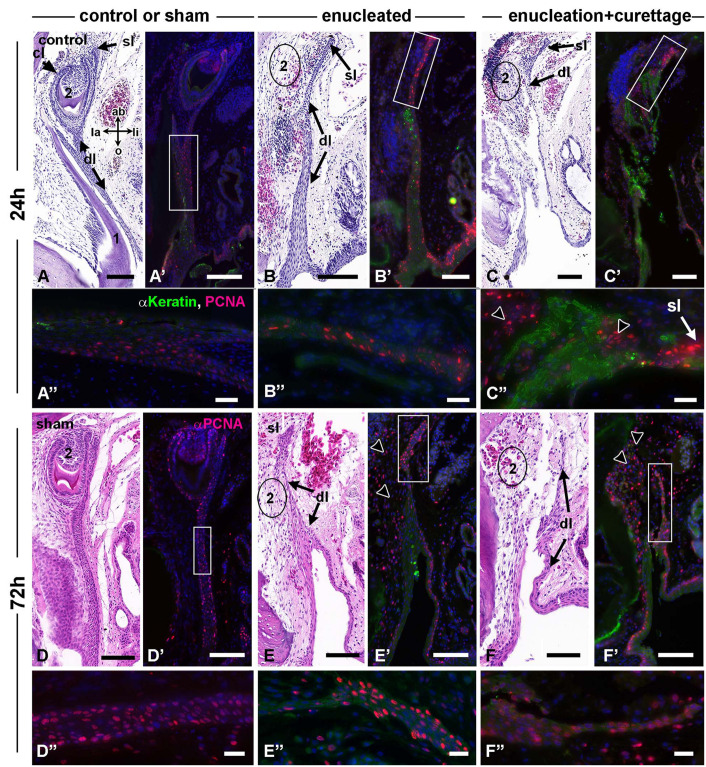
Gecko dental surgical outcomes, 24–72 h. Histological sections stained with H and E and near-adjacent sections stained with PCNA antibody and pan-cytokeratin antibody (Except for **D–D”**). **(A–A”)** The second generation tooth is in advanced bell stage with dentine and enamel deposited. PCNA positive cells are present in the cervical loops, successional lamina, and dental lamina. Anti-cytokeratin labels the epithelium. **(B–B”)** Removal of second generation teeth leaves a space where the tooth used to be (2 with circle around it). The dental lamina is largely intact with proliferating cells concentrated in the successional lamina. **(C–C”)** enucleation and curettage caused tears in the dental lamina. There are shreds of cytokeratin-positive epithelium present **(C’,C”)**. Proliferation is mainly limited to the presumptive successional lamina (black arrowheads, **C”**). After 72 h, healing has begun. **(D–D”)** Sham treated teeth are present and differentiating in the expected location. The dental lamina has many PCNA positive cells **(D”)**. **(E–E”)** The remaining dental lamina continues to have higher proliferation at the aboral tip **(E”)**. There are also large numbers of proliferating mesenchymal cells (black arrowheads). **(F–F”)** Torn dental lamina is starting to heal and there is high proliferation in the epithelium as well as mesenchyme (black arrowheads). Key: ab, aboral; cl, cervical loop; dl, dental lamina; la, labial; li, lingual; o, oral; sl, successional lamina. Scale bars = 100 μm for low power **(A–F’)** and 20 microns for high power images **(A”–F”)**.

By 2 months post-treatment, the control areas show more exclusively orange teeth, indicating they were newly formed since the calcein was administered. Several teeth are still double-labeled and some show only a green calcein-label, indicating that they are no longer actively mineralizing and will soon be replaced (arrows, Figure 2E). The erupted teeth retaining the green label are in tooth positions alternating with orange teeth, and when matched with the developing replacement teeth, waves can be seen in alternating tooth positions increasing in size toward the back of the jaw (Figure 2E, black and white arrowheads). Disruptions to normal eruption patterns are still evident in the regions where teeth were removed (arrows, Figures 2F,G). In the enucleated treatment, newly mineralizing teeth appear in an alternating pattern of large and small teeth (Figure 3F, black and white arrowheads), but do not show a clear pattern of gradual size increase in alternating positions, as seen in the sham control (Figure 2E). In the enucleation with curettage treatment area (Figure 2G), one tooth position shows one functional tooth about to be shed containing green label only, and three partially mineralized replacement teeth following it (white arrow).

Three months post-treatment, every tooth in the sham control is labeled orange, indicating that all teeth have been replaced (Figure 2H). The jawbone is still labeled green (Figure 2H). In the treated areas, teeth are retained that contain a green label only, longer than would be expected when compared to the sham control (Figures 2I,J, arrows). Newly mineralizing teeth in both areas show a pattern of alternating tooth sizes approaching the pattern observed in sham control regions (Figures 2I,J, black and white arrowheads).

### Cellular Responses to Removal of Unerupted, Second Generation Teeth

We demonstrated that after removing the second generation teeth gradual recovery occurred. Next, we examined the tissue-level response. Using a TUNEL assay, cell death is present in the dental lamina and the surrounding mesenchyme 3 days post-surgery in enucleated areas (Supplementary Figures 1A,B). By 7 days post-surgery, there are small amounts of cell death in the mesenchyme but no TUNEL-positive cells in the epithelium (Supplementary Figures 1D,E). The sham control teeth did not have any TUNEL-positive cells. The cell debris was cleared by 1 week.

**FIGURE 4:**
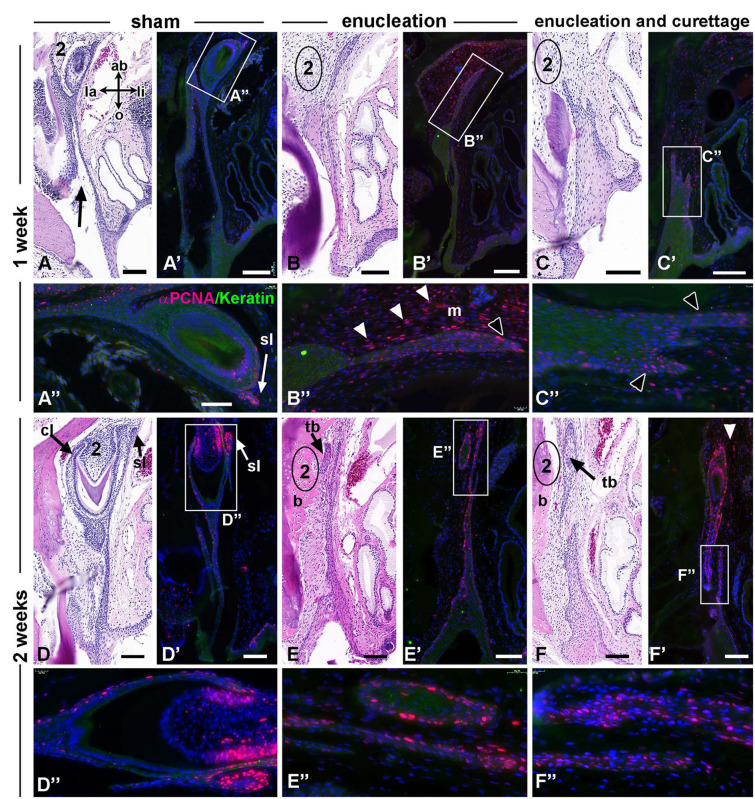
Gecko dental surgical outcomes, 1–2 weeks. Histological sections stained with H and E and near-adjacent sections stained with PCNA antibody and pan-cytokeratin antibody. **(A–A”)** In sham controls, the PCNA positive cells were present in the successional lamina. There is no PCNA staining in the mesenchyme surrounding the tooth or between the dental lamina and bone. Tearing of the dental lamina is still present from the original incision (black arrow). **(B–B”)** The area where the tooth was removed is shown in **(B)** and mesenchyme in this area has increased PCNA staining (arrowheads **B’,B”**). Strong PCNA staining in the free end of the dental lamina is also present (**B”**, black arrowhead). **(C–C”)** The dental lamina is truncated and the cells closest to the torn edge are PCNA positive (arrowheads). **(D–D”)** By 2 weeks the healing of the sham controls is complete and normal PCNA staining is present in the cervical loops and successional lamina. **(E—F’)** In enucleation and enucleation + curettage treatments teeth are beginning to develop at the aboral end. Bone apposition has occurred in the site of tooth removal. The dental epithelium is PCNA positive. Cells in the dental lamina are also positive and this is not affected by local separations of the oral and aboral sections of the dental lamina **(F”)**. Mesenchymal proliferation is present around the tooth buds (white arrowheads). Key: 2, second generation tooth; ab, aboral; b – bone apposition; cl, cervical loop; dl, dental lamina; la, labial; li, lingual; m, mesenchyme; o, oral; sl, successional lamina; tb, tooth bud. Scale bars = 100 μm for low power images and 50 μm for **(A”–F”)**. Bar in **(A”)** applies to all others.

Next, we examined proliferation using PCNA to determine whether the pattern of proliferating cells in the dental epithelium and adjacent mesenchyme was changed by the surgery. The normal pattern of PCNA labeling in bell stage teeth is to have high proliferation in the mesenchyme, cervical loops and successional lamina (Handrigan et al., 2010). The dental lamina typically has much lower proliferation (Figures 3A–A”). Cap and bud stage teeth are 100% labeled with PCNA. During the first 24 h after removal of the second generation tooth the dental lamina retained PCNA-positive cells near the aboral end (Figures 3B–C”). Tearing of the dental lamina did not affect the pattern (Figure 3C”). The sham control appeared to have increased PCNA staining in the dental lamina at 72 h compared to 24 h (Figures 3D–D”). The clearest change between 24 and 72 h was the increase in PCNA label in the mesenchyme in the area where the second generation tooth had been removed (Figures 3E’,F’). The most aboral extension of the dental lamina continued to express PCNA (Figures 3E”,F”). After 1-week, proliferation in the sham controls was similar to unmanipulated dental tissues (Figures 4A–A”). Mesenchymal proliferation was increased next to the location where the second generation teeth were removed (Figures 4B–B”). In areas where the dental lamina was torn, proliferation was noted near the edges of the epithelium (Figures 4C–C”).

Two weeks after surgery, sham controls continued to have strong signal in the second generation teeth mainly in the cervical loops and successional lamina (Figures 4D–D”). The mesenchymal response had changed and there was very little PCNA staining next to the dental lamina (Figures 4E’,E”). New bone was visible on the dental process of the maxillary bone (Figures 4E,F). The dental lamina continued to express PCNA near the aboral end and occasionally new tooth buds could be seen (Figures 4F–F”). The dental lamina was torn in some locations following curettage (Figures 4F–F” and Supplementary Figures 2D,E) but this did not affect proliferation near the aboral tip of the dental lamina. Other phenotypes in the dental lamina are observed following surgical disruption. These include cysts forming within the dental lamina (Supplementary Figures 2A–C), tearing or truncation of the dental lamina (Supplementary Figures 2D,E), thickening or twisting of the dental lamina (Supplementary Figure 2F), and rotation of developing teeth (Supplementary Figures 2G,H). In summary, after tooth removal in the non-curetted regions, the dental lamina remains full-length, extending to the palatine process of the maxillary bone or lingual process of the dentary (Figures 3B,E and Supplementary Figure 2A,B). There was no evidence of retraction of the dental epithelium in the sham surgical sites. We did not have a specific marker for the successional lamina but it seemed likely that that plucking of the mineralized tooth likely brings with it any attached successional lamina (as in Figures 4A,D).

**FIGURE 5:**
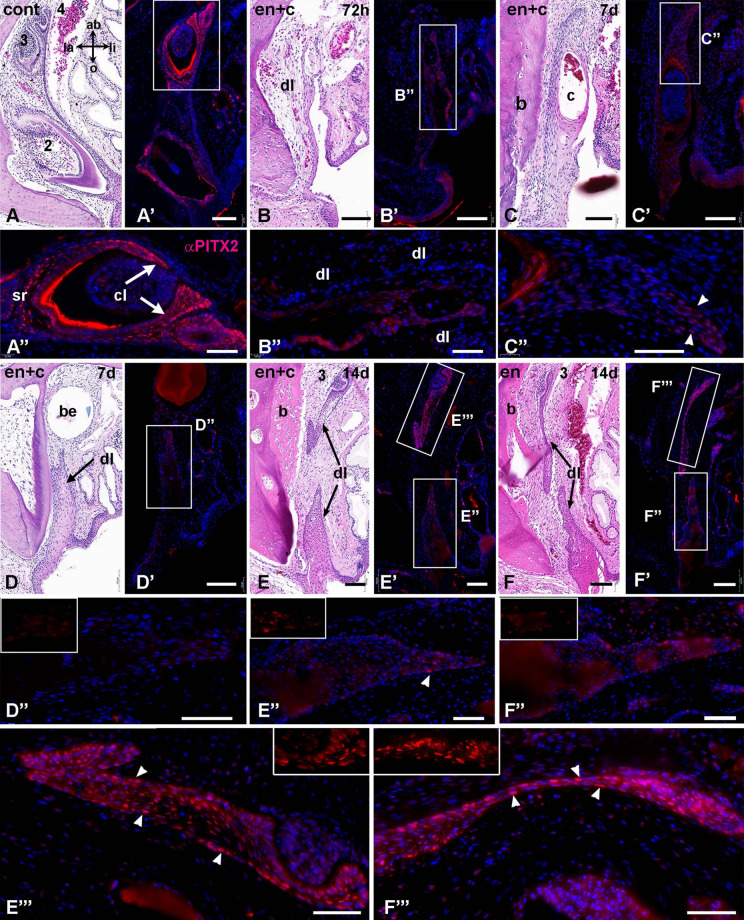
Expression of PITX2 in non-treated and treated dental epithelium. Transverse sections are stained with H and E and near-adjacent sections stained with anti-PITX2 antibody (red) and Hoechst nuclear stain (blue). **(A–A”)** Premaxillary tooth family with three successional teeth. The dental epithelium of the enamel organs is PITX2-positive. **(B–B”)** 72 h after the surgery, the dental lamina retains weak PITX2 staining. **(C–C”**) By 7 days the dental laminae have cysts in some locations. PITX2 nuclear staining is only visible at the most aboral end (**C”**, arrowheads). **(D–D”)** One week post surgery, In areas where the dental lamina is short, there is very weak nuclear staining of PITX2 (**D”** and inset shows PITX2 channel). This section includes the bead which marks the start of the surgical site. **(E–E”’)** Two weeks after enucleation and curettage, bone apposition is present close to where the tooth was removed. There is a separation between oral and aboral sections of the dental lamina. The oral portion has a few cells near the torn edge with PITX2 staining (**E”** and inset). In the aboral dental lamina a tooth bud is beginning to form from the remnants of dental lamina and strong nuclear staining is visible (**E”’**, arrowheads and inset). **(F–F”’)**. There is separation between oral and aboral sections of the dental lamina. The strongest nuclear staining is in the nascent tooth bud and aboral end of the dental lamina (arrowheads in **F”’** and inset). Key: 2, second generation tooth; 3, third generation tooth; 4, fourth generation tooth; ab, aboral; b, bone; be, bead; c, cyst; cl, cervical loop; cont, control; dl, dental lamina; en, enucleation; e + c, enucleation and curettage; la, labial; li, lingual; sl, successional lamina; sr, stellate reticulum. Scale bars = 100 μm for low magnifications and 50 μm for high magnifications **(A”–F”**,**E”’,F”’)**.

We then examined expression of PITX2, a marker of odontogenic potential, in normal tooth development and in tissues with dysmorphic dental laminae. In control tissues, PITX2 protein is expressed in the enamel organ, cervical loops, and successional laminae of developing teeth at all stages (Figures 5A–A”). In contrast, in the other regions of the dental lamina there were very few positive cells (10% of cells in the oral third were PITX2 positive, *N* = 22 as compared to 60% of cells close to the tooth, *n* = 35). Immediately after surgery, there is little expression of PITX2 in the torn dental lamina (Figures 5B–B”), except for the most aboral extension in some cases (Figures 5C–C”). At 1 week, a similar low level of signal was seen in the oral portions of the shortened dental lamina (Figures 5D–D”). By 2 weeks, only pieces of dental lamina that were located close to the typical position of the third generation tooth bud showed initiation of new teeth, even though shortened oral portions had some cells with PITX2 expression (Figures 5E–F”’). Thus, dental identity was maintained in the aboral dental epithelia even though spatial information may have been scrambled. We hypothesized that epithelial-mesenchymal interactions needed to initiate teeth are only supported in specific locations deep in the jaw mesenchyme.

We examined a second label of dental epithelium and one that identifies putative stem cells. SOX2 marks progenitor cells in some mammals (Juuri et al., 2012) and reptiles (Juuri et al., 2013; Kim et al., 2020), sharks (Martin et al., 2016), and bearded dragon (Salomies et al., 2019). Unlike these other animals, and unlike expression detected in embryonic geckos (Juuri et al., 2013) or juvenile geckos (Kim et al., 2020), there was no SOX2 antibody staining in the tooth bud or successional lamina (Supplementary Figure 3B in this 1 week mandibular control section). The differences between our studies may be related to the ages of the animals. Animals used for SOX2 staining here were adults and not juveniles or embryos. There is minimal expression in the dental lamina, with light staining of some nuclei near the oral cavity. There is strong staining in the taste buds, showing that the antibody did cross-react with gecko SOX2 protein (Supplementary Figure 3C). The SOX2-positive cells in the taste buds were complementary to PCNA-positive cells (Supplementary Figure 3C), as reported previously in the mouse (Castillo-Azofeifa et al., 2018). Similarly, in the tooth there is no overlap between PCNA-positive and SOX2-positive cells. There are some regions of the dental lamina where overlapping staining is visible (Supplementary Figures 3E,E’).

We also examined the expression of SOX2 1–2 weeks post surgery (Supplementary Figures 4A–H”). There are similar patterns of staining 1 week after surgery consisting of light SOX2 staining in nuclei of the central and oral portion of the dental lamina (Supplementary Figures 4A–D”). After 2 weeks we identified SOX2-positive cells in the oral portion of the dental lamina and sometimes in the aboral dental lamina. These qualitative observations do not support a change in the number or location of SOX2-positive cells after surgery. These animals were not labeled with BrdU so no direct comparison between SOX2-positive and BrdU-positive cells is possible.

### BrdU Pulse-Chase of Dental Epithelial Cells to Trace the Progeny of Label-Retaining Cells

**FIGURE 6:**
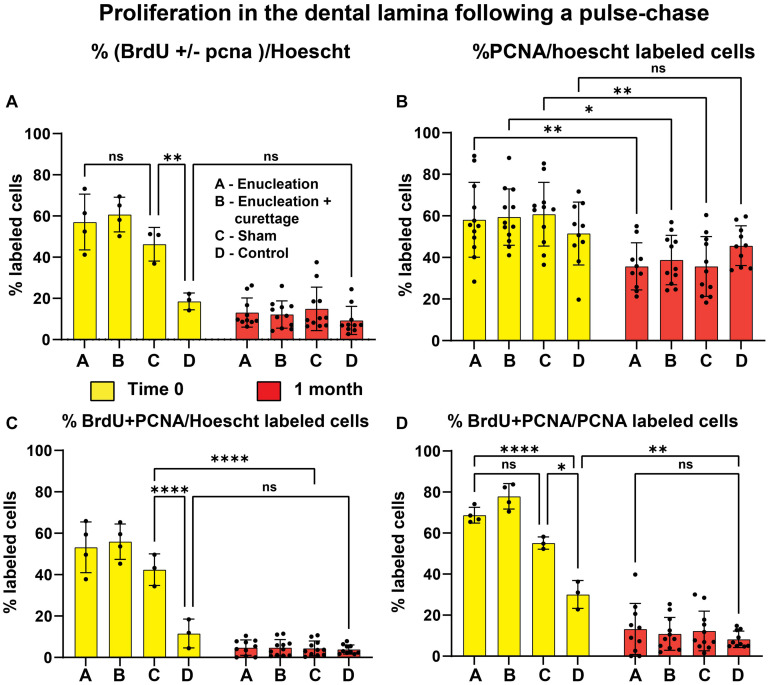
Proliferation in the dental lamina following a pulse-chase. **(A)** The incorporation of BrdU +/- PCNA relative to the total number of cells in the dental lamina was significantly higher in the treated and sham control compared to the non-manipulated controls at time 0. At 1 month there were no significant differences between the types of manipulation. In addition, the number of labeled cells was the same in controls as at time zero. **(B)** PCNA labeling was significantly higher in the time 0 relative to the 1-month post-surgery dental lamina with the exception of control dental laminae. **(C)** At time 0, dual labeled cells were present in significantly higher proportions between the controls and all experimentally treated tissues. There was no significant difference between the percentage of dual labeled cells at time 0 and 1 month in the controls. Other treated tissues had significantly lower labeling at 1 month compared to time 0. **(D)** The proportion of BrdU + PCNA/PCNA cells was very high in the treated tissues at time 0 compared to controls. In addition, there were higher numbers of labeled cells in controls at time 0 compared to 1 month post-chase. Asterisks: *P < 0.05, **P < 0.01, ***P < 0.001, ****P < 0.0001.

**FIGURE 7:**
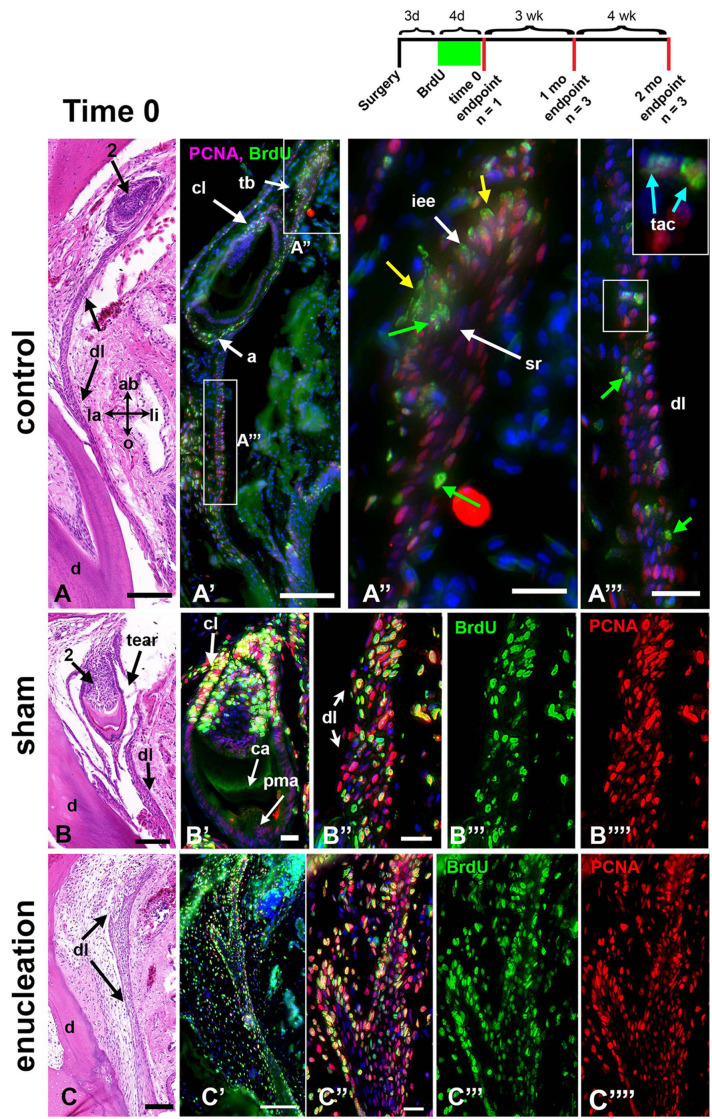
Maximum labeling from 4-day pulse of BrdU – time 0. The experimental paradigm is illustrated above. Three days post-surgery the BrdU pulse begins and carries on for 4 days. Animals are euthanized after the pulse at time 0, 1, or 2 months. Sections were stained with H and E and near-adjacent sections were used for dual labeling with BrdU (green) and PCNA (red). **(A–A”’)** Control, non-manipulated maxillary tooth after a 4-day pulse of BrdU. The ameloblasts that were proliferating took up the label **(A’)** as did the cervical loops and successional lamina **(A”)**. The least mature tooth bud is captured in the near-adjacent fluorescent section **(A”)**. Much of the inner enamel epithelium is dual labeled (yellow arrows). Almost no BrdU is taken up in the stellate reticulum. **(A”’)** The dental lamina is mainly labeled with PCNA but a few cells labeled only with BrdU are also present (green arrows). There are also some dual-labeled transit amplifying cells (blue arrows). The red spot is non-specific stain. **(B–B””)** A sham tooth that was undergoing cytodifferentiation at the time of the label. **(B’)** The original calcein label is visible. Post-mitotic ameloblasts were unlabeled whereas the cervical loops and successional lamina were heavily labeled. **(B”)** The dental lamina contained many more BrdU positive cells than the control **(B”’)** and many of the cells were dual labeled with PCNA (compare **B”’ to B””**). **(C–C””)** Removal of a tooth has resulted in strong labeling of BrdU throughout the dental lamina. Split channels show that many of the PCNA cells are co-labeled with BrdU. Key: 2, second generation tooth; a, ameloblasts; ab, aboral; ca, calcein; cl, cervical loop; d, dentine of first generation tooth; dl, dental lamina; iee, inner enamel epithelium; la, labial; li, lingual; pma, post-mitotic ameloblast; o, oral; sl, successional lamina; sr, stellate reticulum; tac, transit amplifying cell; tb, tooth bud. Scale bars = 100 μm in **(A,A’,B,C,C’)**. All other bars = 20 μm.

**FIGURE 8:**
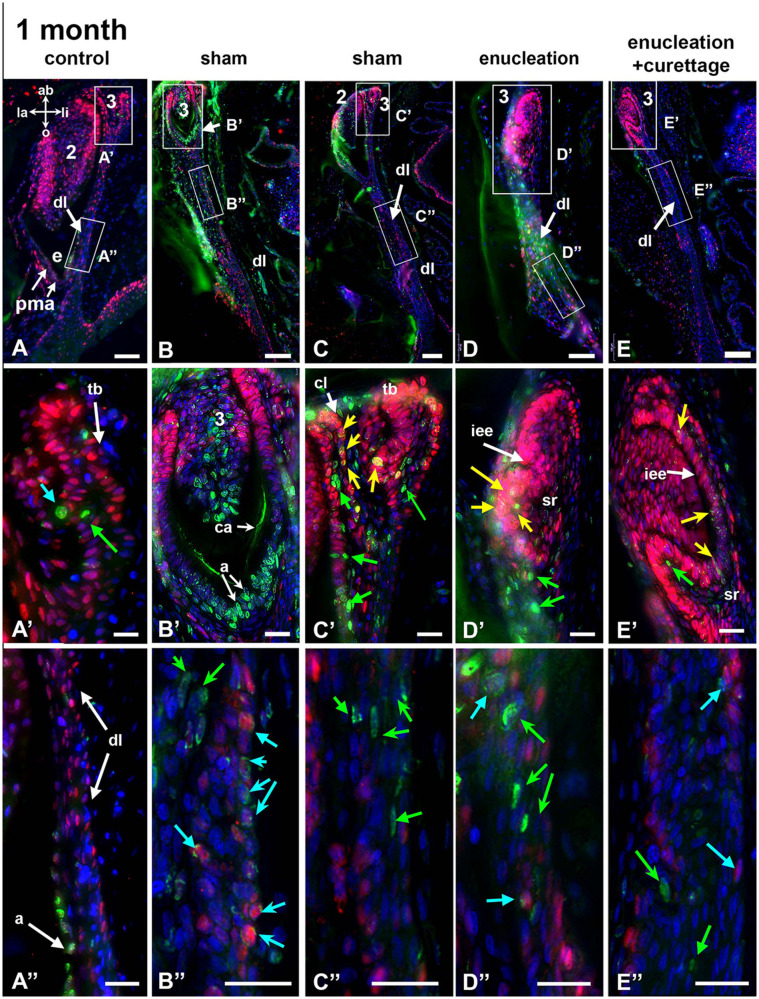
BrdU label retention after 1 month and co-labeling with PCNA. Five teeth **(A–E)** sampled from different regions of the same animal. BrdU is green, PCNA is red, and nuclei are blue. Axes in A apply to all panels. **(A–A”)** A tooth family with BrdU retained in some cells within the third generation tooth bud **(A’)**. Most of the dental lamina is labeled with PCNA but not BrdU **(A”)**. The ameloblasts formed at the time the label was administered have taken up BrdU **(A”)**. **(B–B”)** The second generation tooth bud has been labeled with calcein and the ameloblasts are labeled with BrdU. The newest enamel is deposited between the calcein label and cusp tip. The majority of BrdU has been trapped in the post-mitotic ameloblasts and therefore this tooth is about 1 month old. The dental lamina contains many dual-labeled cells that are transit amplifying cells (blue arrows). Several label-retaining cells are only labeled with BrdU (green arrows). **(C–C”)** Many BrdU-labeled cells (green arrows) and dual-labeled cells (yellow arrows) are in the new tooth bud and cervical loop of the second generation tooth. The dental lamina is also labeled with BrdU **(C”)**. **(D–D”)** A new tooth bud that was not present at the time of surgery. There are dual labeled cells in the inner enamel epithelium and stellate reticulum (yellow arrows, **D’**). The dental lamina has multiple BrdU cells (green arrows) and several adjacent transit amplifying cells (blue arrows). **(E–E”)** A third generation tooth bud that has labeled cells in the inner enamel epithelium (**E’**, yellow arrows). The dental lamina has interstitial BrdU labeled cells and a few transit amplifying cells (blue arrows). Key: 2, second generation tooth; 3, third generation tooth; a, ameloblasts; ab, aboral; ca, calcein; cl, cervical loop; dl, dental lamina; e, enamel; iee, inner enamel epithelium; la, labial; li, lingual; o, oral; pma; post-mitotic ameloblast; sr, stellate reticulum; tb, tooth bud. Scale bars = 20 μm for **(A’–E”)**, 50 μm for **(A,D)** and 100 μm for **(B,C,E)**.

**FIGURE 9:**
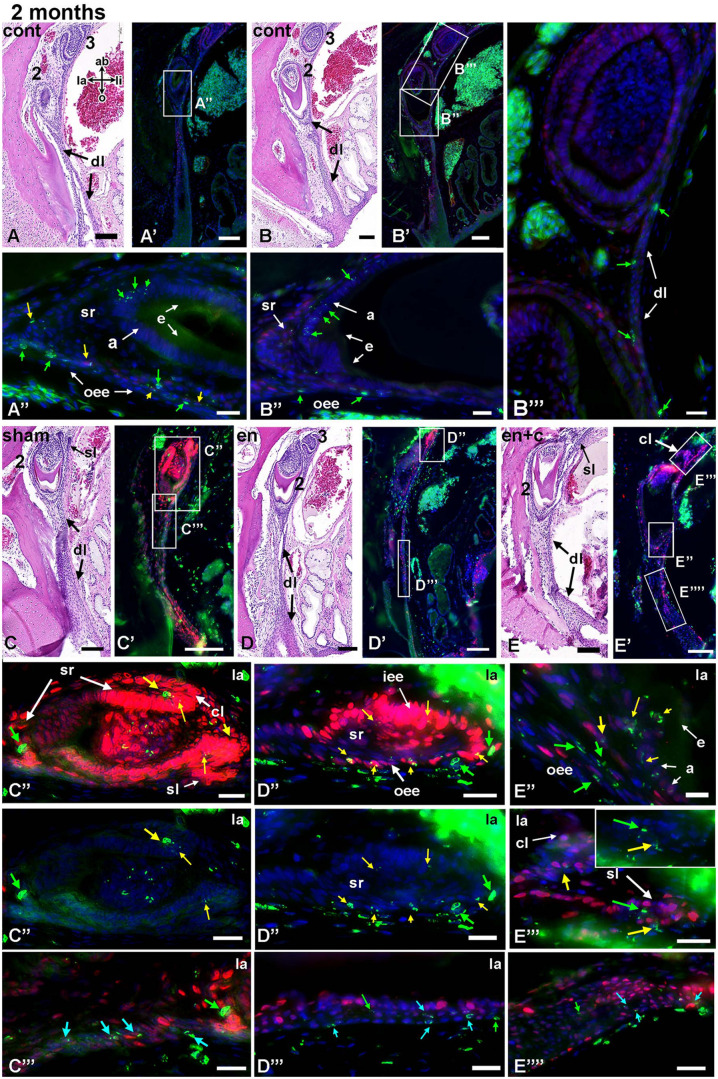
BrdU label retention after 2 months and co-labeling with PCNA. Transverse sections of teeth from different regions of the maxilla stained with H and E. Near-adjacent sections were used for immunofluorescence staining. **(A–B”’)** Second generation teeth have retained fragments of BrdU in the nuclei of the post-mitotic ameloblasts. **(B”’)** Label-retaining cells are present in the dental lamina between the second and third generation teeth (green arrows). **(C–C”’)** A differentiating tooth has BrdU-retaining cells in the stellate reticulum (green arrows). Dual labeled cells are scattered in the cervical loops. **(C”’)** In the dental lamina there are several BrdU and transit amplifying cells (blue arrows). **(D–D”’)** A tooth family with a second generation tooth that was in bud stage at the time of surgery. The newest tooth bud derived from the successional lamina of the second generation tooth and contains dual labeled cells in the inner and outer enamel epithelium. The dental lamina continues to harbor lightly labeled, BrdU-positive cells (green arrows, **D”’**). **(E–E””)** A tooth in bell stage that has just started to deposit enamel matrix. Fragments of BrdU are in the ameloblasts and outer enamel epithelial cells. This tooth was in early bud stage during the original surgery. The successional lamina which arose since the surgery contains BrdU labeled cells (green arrows). Key: 2, second generation tooth; 3, third generation tooth; a, ameloblasts; ab, aboral; cl, cervical loop; cont, control; dl, dental lamina; en, enamel matrix; iee, inner enamel epithelium; la, labial; li, lingual; o, oral; oee, outer enamel epithelium; sl, successional lamina; sr, stellate reticulum. Scale bars = 100 μm for low magnification views and 20 μm for **(A’–E”’)**.

In order to determine whether previously purported stem cells in the dental lamina (Handrigan et al., 2010) give rise to new teeth, we performed a BrdU pulse-chase analysis (Figures 6–9). Based on the time-course of PCNA expression, proliferation in the dental lamina was highest 3–7 days after surgery (Figure 3). Therefore, we began injecting BrdU 3 days post-surgery and continued for 4 days. We anticipated that we would label putative stem cells that may have been activated in response to the injury. We analyzed multiple teeth across three different animals (Supplementary Table 2).

#### Time Zero, Normal Proliferation Patterns and the Maximum Incorporation of BrdU

One gecko was immediately euthanized after the 4-day BrdU pulse (7 days post surgery, Animal 9) to determine the maximum proportion of labeled cells at time 0 under different treatment conditions (Figures 6, 7 and Supplementary Tables 1, 2). We also examined 2 other animals with PCNA labeling only (Animals 5 and 11, Supplementary Tables 1, 2). PCNA would be present in cells that were in late G1, S, and G2 to M transition phase (Kurki et al., 1986) and this label represents the normal proliferation in the tissue at that moment in time. In contrast, BrdU had been sequentially added to the animals over 4 days. Therefore, cells entering S phase on any of the 4 days would be cumulatively labeled. There is insufficient time to dilute the original BrdU label between days 1 and 4.

We were particularly interested to know whether the surgery had caused normally quiescent dental lamina cells to divide and take up BrdU. In the control regions there were less than 20% of dental lamina cells that were BrdU-positive with or without PCNA label (BrdU +/- PCNA; Figures 6A, 7A–A” and Supplementary Table 3A). In contrast, in the treated regions, approximately 50% of dental lamina cells were labeled with BrdU +/- PCNA (Figures 6A, 7B–C”). This level of incorporation was significantly higher than the controls (Figures 6A, *P* < 0.01, Supplementary Table 3A). These results suggest that the retraction of the mucosa in the shams stimulates incorporation of BrdU. We also assessed the number of cells that just had BrdU label at time 0 (Supplementary Table 2, and Supplementary Figure 5). These are cells that are not in the cell cycle as shown by lack of PCNA staining. The BrdU-positive cells may have been stimulated to divide immediately after the surgery but prior to euthanasia the cells had entered G0 or early G1 and hence were PCNA negative (Kurki et al., 1986).

In the control and sham control regions, all terminally differentiated, post-mitotic ameloblasts that were present before the surgery were unlabeled (Figure 5B’). The calcein label visible in the mineralized dentine or enamel distinguishes teeth that were present at the time of the surgery (Figure 5B’).

The percentage of PCNA labeling in the dental lamina represents cells in the cell cycle at the time of euthanasia. There was no difference across the treatments and controls at time 0 (Figures 6B, 7A”’–C”’ and Supplementary Table 3B). From these data we cannot rule out a change in the basal level of proliferation compared to an animal that did not have surgery.

We then calculated how efficiently we had labeled cells that were in the cell cycle with BrdU. At first, we looked at the proportion of dual-labeled cells out of the entire population of cells in the dental lamina (BrdU + PCNA labeled, Figure 6C). There were significantly more cells labeled compared to control dental laminae (Supplementary Tables 3A,C and Figures 6C, 7B”,C”). In a related analysis, the proportion of BrdU + PCNA labeled cells out of all the PCNA positive cells was higher (70–78%, Figure 6D and Supplementary Tables 3A,D) than in control dental laminae. In summary, we had identified a population of single-labeled, BrdU-positive cells in the dental lamina that were not in the cell cycle. These could therefore be putative progenitor or stem cells. The surgery did not affect the proportion of these slower-dividing cells. However, the surgery did significantly increase the number of BrdU-labeled cells entering the cell cycle (BrdU + PCNA).

#### One-Month Post-surgery

We examined three animals 3 months after surgery (Figure 8 and Supplementary Figure 6). By 1 month post-surgery it was hard to find torn dental laminae therefore it appears that they had healed completely (Supplementary Figures 2G,H,5D,E). We are unable to determine whether healing occurred from dental lamina growing in from the edges of the defect, whether there was aboral growth of the shorter, torn dental lamina, or a mixture of both. Typically an erupted tooth will remain in place for approximately 5–8 weeks while it is in function (Figures 2E,H; Grieco and Richman, 2018). Then the second generation tooth erupts and 4 weeks elapse before the third generation tooth erupts (Figure 2H). Thus, 1 month after removing the second generation teeth we estimate that third generation teeth have likely progressed to cytodifferentiation. There will be some teeth that formed since the surgery that were not initially present. These would have reached cap stage but no ameloblasts would have differentiated. In sham or non-manipulated controls, we would see the second generation tooth has formed the full crown and is resorbing the 1st generation tooth.

The BrdU label serves two purposes 1-month post-surgery. The first is to differentiate putative stem cells from transit amplifying cells in the dental lamina. The most slowly dividing, undifferentiated dental lamina cells will retain most of the single BrdU label (the nucleus will be filled with the fluorescence signal) and importantly, will not be labeled with PCNA. The dental lamina cells that are dual labeled with fragmented BrdU and with PCNA are likely to be transit amplifying cells. The second purpose is to differentiate between three types of teeth: (1) those that formed from an existing tooth bud present at the time of surgery, (2) those that intiated immediately after surgery, and (3) those that were formed *de novo* several weeks after surgery from remaining dental lamina. The presence of terminally differentiated, post-mitotic unlabeled ameloblasts combined with the calcein label (visible under UV illumination of the sections) indicates the first tooth type, a tooth bud that was present and partially differentiated prior to conducting the surgery. The second type of tooth will have ameloblasts that are fully labeled with BrdU but became post-mitotic almost immediately after surgery. The *de novo* or third type of teeth will have reached cap or bud stage, will be strongly labeled with PCNA, and will have a proportion of cells also labeled with fragmented BrdU. Note that xylenol orange was present in the specimens but was not retained following processing for histology.

In non-manipulated controls, large second generation teeth were present in the expected location. As predicted, the majority of ameloblasts did not pick up the BrdU because they were post mitotic (Figure 8A and Supplementary Figure 6A) however the least mature ameloblasts were labeled and then ceased dividing (Figure 8A” and Supplementary Figure 6A”). Third generation teeth were present ranging from bud to bell stage. In bud stage teeth there were very few BrdU positive cells (Figure 8A’). The dental lamina was very weakly labeled (Figure 8A”). The same pattern was observed in sham controls (Figure 8B). Third generation teeth that were present at the time of surgery could easily be distinguished by the presence of calcein label and the heavily labeled, secretory ameloblasts (Figures 8B,B’ and Supplementary Figures 6B,B’). Tooth buds in the sham areas that had not been present originally were labeled in all areas of the enamel organ (Figures 8C,C’ and Supplementary Figures 6C,C’). In the areas where second generation teeth were removed, the most aboral teeth close to the palatine process of the maxillary bone were in bud or cap stage (Figures 8D–E’). These teeth were lightly labeled with fragmented BrdU in the inner enamel epithelium which was also PCNA positive (Figures 8D–E’). These dual labeled cells in the least mature teeth raised the possibility that the original BrdU labeled cells in the dental lamina gave rise to all the layers of the enamel organ of the new teeth.

Transit amplifying cells are the first progeny of a stem cell to form after an asymmetric cell division. As such, transit amplifying cells should retain much of the original BrdU label and be in close proximity to the stem cell niche (Yu and Klein, 2020). The dental laminae of sham controls and treated teeth had label retaining cells plus several transit amplifying cells close by (Figures 8B”–E”).

Our quantification of proliferation in the dental lamina at 1 month across three animals found that percentage of BrdU +/- PCNA labeled cells and other related calculations had significantly dropped compared to time zero (Figures 6A,C, 8A–E”). There was no significant difference in the control teeth between time 0 and 1 month (Figures 6A,C, 8A” and Supplementary Table 4A). Interestingly there was no significant difference in the proportion of exclusively BrdU-labeled cells between time 0 and 1 month (Supplementary Figure 5). The numbers varied quite a lot between sections so statistical significance was not reached (Supplementary Table 2 and Supplementary Figure 5). Nevertheless, in the majority of sections, the dental lamina had retained quite a few putative stem cells (Figures 8B”–D”,E’). The proportion of BrdU + PCNA/PCNA cells dropped significantly between time 0 and 1 month (Figure 6D). A part of the difference was due to the decrease in overall BrdU labeling at 1 month (Figure 6B). The PCNA data from the dental lamina suggests that at time 0 the animal did respond to the surgery by an overall increase in proliferation (possibly a general stress-response) but that by 1 month, homeostasis was restored. The level of PCNA labeling seen in the controls at time 0 was not significantly different than controls at 1 month and therefore represents the typical level of proliferating cells in the dental lamina.

The teeth that were present in bell stage at the time of surgery, no matter what the type of treatment, often had calcein labeling but ameloblasts at the cusp tip were unlabeled because they were post-mitotic (Figures 8A,C). Teeth that were in cap or early bell stage at the time of surgery had taken up BrdU in the ameloblasts and then stopped dividing (Figure 8B’). As predicted, teeth that were either not present or in early bud stage had progressed either to bud or cap stage but not to bell stage. The enamel organs and successional laminae of these teeth had strong PCNA labeling but also there were some BrdU positive cells present (Figures 8A’,C’–E’). A similar pattern of staining in the successional lamina (strong PCNA with a minority of cells labeled with BrdU) was reported by others (Kim et al., 2020). We did not quantify the proportion of dual labeled cells in the teeth at 1 month post surgery because it was highly variable.

#### Two Months Post-surgery

We examined three animals, 2 months after surgery. Regrettably, two of the animals did not have sufficient penetration of the fixative so the majority of teeth originated from 1 animal (Figures 9A–E””, Supplementary Figures 7A–B”’, and Supplementary Table 4).

Based on the timing of tooth succession, by 8 weeks the tooth buds that were in bud or cap stage at the time of surgery would have reached position #2 and would have entered late bell or histodifferentiation stage (Figures 9A–E). Tooth buds that were not present at the time of surgery would be in early cap stage (Figures 9A,B,D). The bell stage teeth whether in control or treated tissues had BrdU in the inner enamel epithelium (Figures 9A”,B”,E”). There were also fragments of BrdU present in the outer enamel epithelium (Figures 9A”,B”’,C”–E”). The bud stage teeth that were not present at the time of surgery contained labeled cells in the outer and inner enamel epithelium, the majority of which were dual labeled (Figures 9D”,E”’ and Supplementary Figures 7A”,B”). The frequent presence of BrdU +/- PCNA cells within the dental lamina after removal of second generation teeth and the presence of fragmented BrdU in the new tooth buds suggests that the dental lamina is contributing to the next generation of teeth. The dental lamina retained BrdU-positive cells in all treated and control tissues (Supplementary Table 4 and Figures 9C”’,D”’,E””). There were cells labeled with PCNA and BrdU and based on their location in the dental lamina, they could be transit amplifying cells. A proportion of dental lamina cells were only labeled with BrdU and these are likely the undifferentiated, slowly dividing progenitor cells.

## Discussion

This study transiently disrupted tooth replacement in adult geckos by selectively removing the unerupted, second generation mineralized teeth. The healing of the dental lamina and resumption of tooth replacement is a robust process and was approaching normal patterns by 3 months. Although there were parts of the dental lamina that were torn and the successional lamina was disrupted, the dental lamina retained odontogenic identity, healed, and *de novo* tooth formation occurred. We also present the first data comparing normal and healing dental laminae in adult animals. We also determined that even though teeth were initially forming much closer than normal to each other in disrupted dental and successional laminae, there was no inhibition of development. Once the subsequent generation of teeth initiated, teeth form as independent units in the correct position, deep in the jaws, based on local epithelial-mesenchymal signaling.

### Replacement Timing and Patterning of the Adult Gecko Dentition Is Controlled by Local Rather Than Regional Factors

Our work partially tested the hypothesis that in the adult reptile dentition there are diffusible signals acting between tooth families going over long distances in the jaw to set up the tooth replacement pattern (Edmund, 1969). Work on other animal models suggested that there is a zone of inhibition between nearby teeth, in cichlids (Fraser et al., 2008) and in denticles of shark skin (Cooper et al., 2018) that establish patterning. In our enucleation experiments, mineralizing teeth were removed, but the dental lamina, successional lamina, and any bud-stage teeth that did not show fluorescently labeled mineralizing tissue were left in the jaw. This explains the quick recovery in which teeth that had already initiated maintained the timing and patterning of subsequent tooth initiation. In the curetted treatment sites, some tooth buds were pushed close together or twisted into different positions, yet still continued to develop even though the implied zone of inhibition was disrupted. This suggests that, if present in reptiles, the zone of inhibition is more important pre-initiation for spacing the dentition but less important once teeth begin differentiation. We acknowledge that other experiments are needed to determine what would happen to the pattern of initiation if all the third generation teeth were removed in a localized region.

We noted at two weeks post-surgery that teeth initiated only in the most aboral sections of the dental lamina. The instructive signals to begin tooth formation may lie either in the mesenchyme or epithelium. Currently we lack markers of odontogenic mesenchyme and have no method of separating the two tissues *in vivo*. Potential molecules that may be involved in local patterning in the reptile dentition were reported in a study on the bearded dragon (Salomies et al., 2019). These authors performed bulk RNA sequencing specifically on the successional lamina and surrounding mesenchyme using tissues isolated with laser capture microdissection. Some candidate mesenchymal genes were presented but many of these are not annotated. More bioinformatics work needs to be done to find potential instructive signaling pathways involved in tooth induction in adult reptiles.

### Signals From Unerupted Teeth May Be Needed to Correctly Time Tooth Shedding

Our data suggest that the dental lamina contains the information to correctly pattern newly initiating teeth, and signals are not emanating from the second generation tooth. However, the second generation tooth may be required for the resorption of the functional tooth. We found that the functional tooth was retained in the mouth for longer than expected and was not replaced until a replacement tooth was present to resorb it. The importance of tooth resorption in maintaining tooth cycle timing has also been shown in studies where the functional tooth was broken, but not removed, in fish (Huysseune et al., 2012) and iguanas (Brink et al., 2020). The timing of tooth replacement in iguanas was not affected by breakage of the tooth crown, since the base still needed to be resorbed before being shed. The gecko teeth appear to require signals from the replacement tooth to initiate resorption of the functional teeth.

### Timing of Initiation of Replacement Teeth Is Not Accelerated Following Tooth Enucleation

Besides affecting tooth resorption, our data do not show evidence for earlier initiation of teeth after enucleation of replacement teeth. Tooth buds that were present at the time of surgery developed normally after the treatment, as shown by the presence of small mineralized teeth in μCT scans. A similar presence of third generation teeth in early stages of histodifferentiation was seen in histology. Perhaps the best evidence for a lack of acceleration in the rate of tooth development is in the PCNA data. After 1 month there was a decrease rather than an increase in PCNA labeling in the dental lamina across all conditions including controls. The teeth themselves are labeled with PCNA in a similar pattern at time 0, 1, and 2 months. The new teeth, once they initiate morphogenesis, are highly proliferative and this is not dependent on whether second generation teeth were removed.

Our results differ from results in the alligator, where removal of a functional tooth triggered proliferation and initiation of new teeth earlier than expected (Wu et al., 2013). The long-term effect of tooth removal in alligators has yet to be reported. Thus, it is not known whether the proliferation ultimately affects the formation of teeth or whether the same group of cells becomes quiescent for a period of time. In the green iguana, proliferation of the successional lamina occurs only when a tooth initiates, so there are much longer periods of quiescence between initiation events than in the leopard gecko (Brink et al., 2020). In the gecko, there is always a new tooth budding at most tooth positions and all successional laminae are proliferating as shown by PCNA labeling. This readiness to form new teeth may explain the more rapid turn over of teeth in the gecko compared to the iguana (4–5 weeks in geckos, Grieco and Richman, 2018; compared to 10–20 weeks in iguana, Brink et al., 2020).

### BrdU-PCNA Labeling Distinguishes Putative Stem Cells From Their Progeny by Location and BrdU Fragmentation

The retention of BrdU label was first reported in the dental lamina, close to the second generation teeth (Handrigan et al., 2010). In that study there was retention of labeled cells in the undifferentiated dental lamina and in the outer enamel epithelium of differentiating teeth. It was also previously reported that there was uptake of BrdU in ameloblasts that were about to differentiate and become post-mitotic (Handrigan et al., 2010). In the present study, we combined PCNA and BrdU labeling, which added to the story. First, with higher resolution, we were able to see that the retention of BrdU varies in quality (extent of fragmentation) and quantity (number of cells) according to the location and stage of differentiation of the tooth buds. Second, we could distinguish cells that were carrying BrdU that had likely entered G0 from those that had entered the cell cycle (BrdU + PCNA). In the previous study, cell counts included BrdU +/- PCNA labeled cells. Some of the cells were likely transit amplifying cells and not exclusively BrdU label-retaining cells. In the previous study it was hypothesized that there would be progeny of the transit amplifying cells in the new tooth buds, ultimately contributing to the different layers of the enamel organ. Here we found that removal of second generation teeth allowed us to separate the contributions of label-retaining cells in the dental lamina from those that may be present within the tooth itself.

### Two Interpretations of BrdU-Labeled Cells – Dedifferentiation Versus Stem/Progenitor Cells

A recent study has suggested that under conditions of chemical ablation, the partially differentiated cells of the inner enamel epithelium in the mouse incisor may revert to progenitor cells (Sharir et al., 2019). This idea that tissues can regenerate even if the local stem cell population is almost depleted has been demonstrated in the intestine and other organs (Gola and Fuchs, 2021; Shivdasani et al., 2021).

We feel it is unlikely that BrdU-positive cells in the dental lamina of the gecko were terminally differentiated and then in response to injury had dedifferentiated into progenitor cells. For the first part of the hypothesis to be true, the dental lamina would need to have characteristics of a differentiated epithelium. There are no signs of stratification as in oral non-keratinized epithelia (Groeger and Meyle, 2019). Since the epithelial cells near the basement membrane are proliferative and undifferentiated in all stratified epithelia (Gola and Fuchs, 2021), any post-mitotic, terminally differentiated cells would be confined to the center of the dental lamina. However, in the gecko and bearded dragon, BrdU-positive cells are located close to the basement membrane often nearby to dual labeled cells. In a study from a different lab (Kim et al., 2020) sections of the leopard gecko dentition were stained with antibodies to the basement membrane protein laminin5. The expression in the successional lamina was diffuse and did not form a distinct outline of the epithelium as in the mouse. This is consistent with the successional lamina not being a differentiated epithelium. The structure of the basement membrane around the dental lamina has yet to be examined. The dental lamina does not serve a barrier function like other oral epithelia and thus does not need specialized cell junctions at the surface. In addition, we showed that the expression of PITX2 is restricted to epithelia that are committed to an odontogenic lineage, whereas most of the dental lamina has lower numbers of PITX2-positive cells. Had dedifferentiation been a major mechanism in the formation of new teeth, we would have seen loss of PITX2 staining in the aboral parts of the dental lamina.

The concept that the dental epithelium had dedifferentiated would be supported by an increase in stem cell marker expression. However, SOX2 is not expressed in most of the gecko dental lamina where BrdU-labeled cells were often found. In addition, there was no increased signal for SOX2 following surgery. Further co-localization experiments will be done to verify the expression of SOX2 and other stem cell markers with respect to the label-retaining cells in the gecko.

It is interesting that immediately following injury, there was an increase in the number of BrdU + PCNA cells. This suggests that the surgery had stimulated more cells that were originally quiescent to enter the cell cycle. However, the number of BrdU-only labeled cells did not change between the original time 0 and 1 month. The gecko dentition must have a means to maintain a minimum number of stem cells throughout life.

### Dual Labeling With PCNA and BrdU Separates Transit Amplifying Cells From Adult Tissue Stem Cells

The transit amplifying cells play an important role in regenerating the dentition. The traditional view is that transit amplifying cells are close to the stem cells but lack the markers of epithelial stem cells such as Lgr5 and Sox2 (Binder et al., 2019). Transit amplifying cells are also proliferative (Yu and Klein, 2020). The strongest evidence that these dual labeled cells in the dental lamina are TA cells comes from the surgical design of the study and the fact that only dental lamina or immature tooth buds were left after removal of second generation teeth. The remaining tooth buds, seen at 1 month of age as cap stage teeth, were highly proliferative and contained fragmented BrdU. In contrast, the main location with nuclei that were fully labeled with BrdU was the dental lamina.

In the present study we were able to accurately identify nuclei that either had pure BrdU label, pure PCNA label, or both. The dual-labeled cells were present in many different locations in the dental epithelium but based on the work of others in the mouse incisor (Harada et al., 1999) and hair follicle (Hsu et al., 2014; Yang et al., 2017), only those dual labeled cells close to the stem cell niche should be considered as transit amplifying cells. In contrast, the undifferentiated epithelial cells (not ameloblasts) that were solely labeled with BrdU were almost always located in the dental lamina, except for a few occurrences in the stellate reticulum. These BrdU-labeled cells were likely the equivalent of adult tissue stem cells found at the base of hair follicles (Yang et al., 2017; Gola and Fuchs, 2021).

Our work does not identify a consistent labio-lingual pattern where previously we reported that most of the BrdU-labeled cells were on the lingual surface of the dental lamina (Handrigan et al., 2010). We attribute this difference to the fact that we sampled many more teeth in the present study and our imaging techniques were more advanced. In addition, some of the lingual cells in the previous study would have been dual labeled but were not identified as such.

Our new model is that some of the transit amplifying cells located in the dental lamina move or are displaced toward the budding teeth. Indeed, we often found clusters of dual-labeled cells very close to the tooth within the dental lamina. The movement of cells from the oral to aboral parts of the dental lamina was also observed in the bearded dragon (Salomies et al., 2019). This directional flow of cells in the dental lamina may direct tooth budding to deeper rather than superficial regions of the jaw.

There are very few models in which to study adult dental stem cells, with the exception of the mouse incisor and teeth in certain reptiles. The mouse has the obvious advantage of being able to perform lineage tracing with genetic markers. The proxy for lineage tracing used here was the extent of dilution of the BrdU label and location of the progeny. We showed that BrdU-labeled cells resident in the dental lamina are generally more fully labeled with BrdU than the highly fragmented BrdU-labeled cells in the tooth germs. Thus BrdU-labeled cells can maintain a subset of progeny with less frequent cell divisions while other subsets are dividing more rapidly and giving rise to the teeth. These BrdU-labeled cells appear to be multipotent. The adult leopard gecko does have desirable properties for understanding dental epithelial stem cells. There is continuous tooth replacement in more than 40 tooth positions per quadrant so there are robust mechanisms to maintain tissue homeostasis amenable to experimental studies. We tested whether removing sections of the dental lamina in a targeted manner (curettage) would delay tooth formation. Remarkably, the integrity of the dental lamina was restored between 2 weeks and 1 month after surgery. There was no delay in forming the replacement teeth as shown on μCT scans. We attribute the rapid healing to the persistence of stem cells in the dental lamina remnants. We predict that a more severe treatment such as chemical ablation (Sharir et al., 2019) may give different results. Taking the normal propensity of the dental lamina to maintain homeostasis, together with the ability of the dental lamina to heal easily and the retention of single BrdU-labeled cells over several months, our data support the idea that the dental lamina houses the dental epithelial stem cell population.

## Data Availability Statement

The raw data supporting the conclusions of this article will be made available by the authors, without undue reservation.

## Ethics Statement

The animal study was reviewed and approved by University of British Columbia Animal Care Committee.

## Author Contributions

KB and JMR: conceptualization, execution of experiments, analysis of data, and drafted the manuscript. TG: conceptualization, execution of experiments, analysis of data, and edited the manuscript. JIH: edited the manuscript, immunostaining, analysis of cell proliferation. JIH and JRMC: proliferation analysis. KF: immunostaining. All authors contributed to the article and approved the submitted version.

## Conflict of Interest

TG is currently employed by the company STEMCELL Technologies. All lab work performed by TG was prior to employment at STEMCELL Technologies. STEMCELL Technologies has no financial or experimental contribution to this work. The remaining authors declare that the research was conducted in the absence of any commercial or financial relationships that could be construed as a potential conflict of interest.
