# Supplementary material for: Tooth Removal in the Leopard Gecko and the de novo Formation of Replacement Teeth
Source: Front Physiol. 2021 May 4;12:576816. doi: 10.3389/fphys.2021.576816 (PMC8126719; doi:10.3389/fphys.2021.576816)
Supplement: Supplementary file 1 [file Data_Sheet_1.pdf]

Supplementary Information

Supplementary Figures

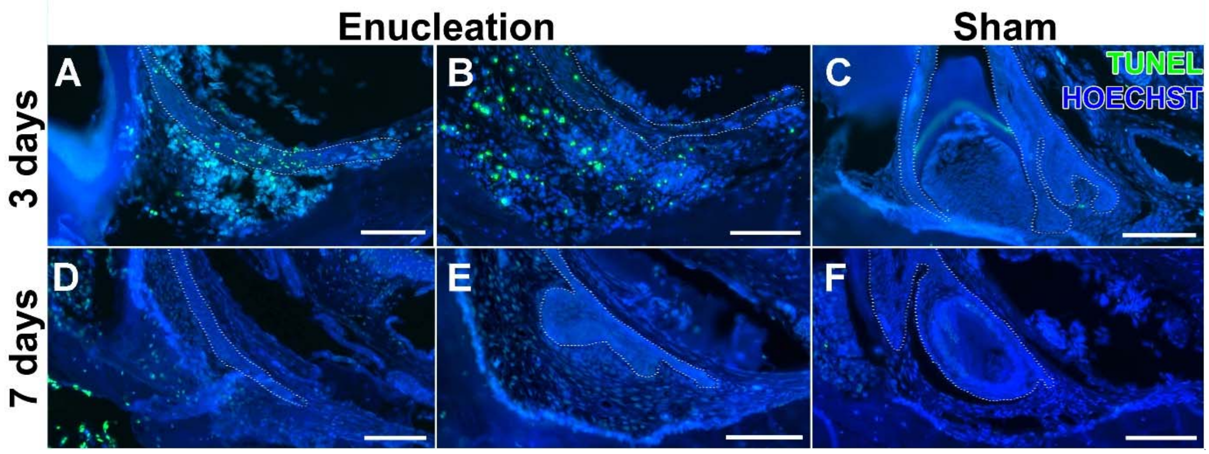

Figure S1: TUNEL assay for apoptotic cells. A-B) cell death is detected in the mesenchyme and dental lamina post-enucleation in two different regions of the mandible. C) No cell death is seen in the tissues within the sham area. D-E) Small amounts of cell death are detected in the mesenchyme, but not in the dental lamina in two different regions of the mandible. F) The sham area of the same mandible is normal, with no cell death detected. White dotted line indicates dental lamina. Scale bars= 100 µm

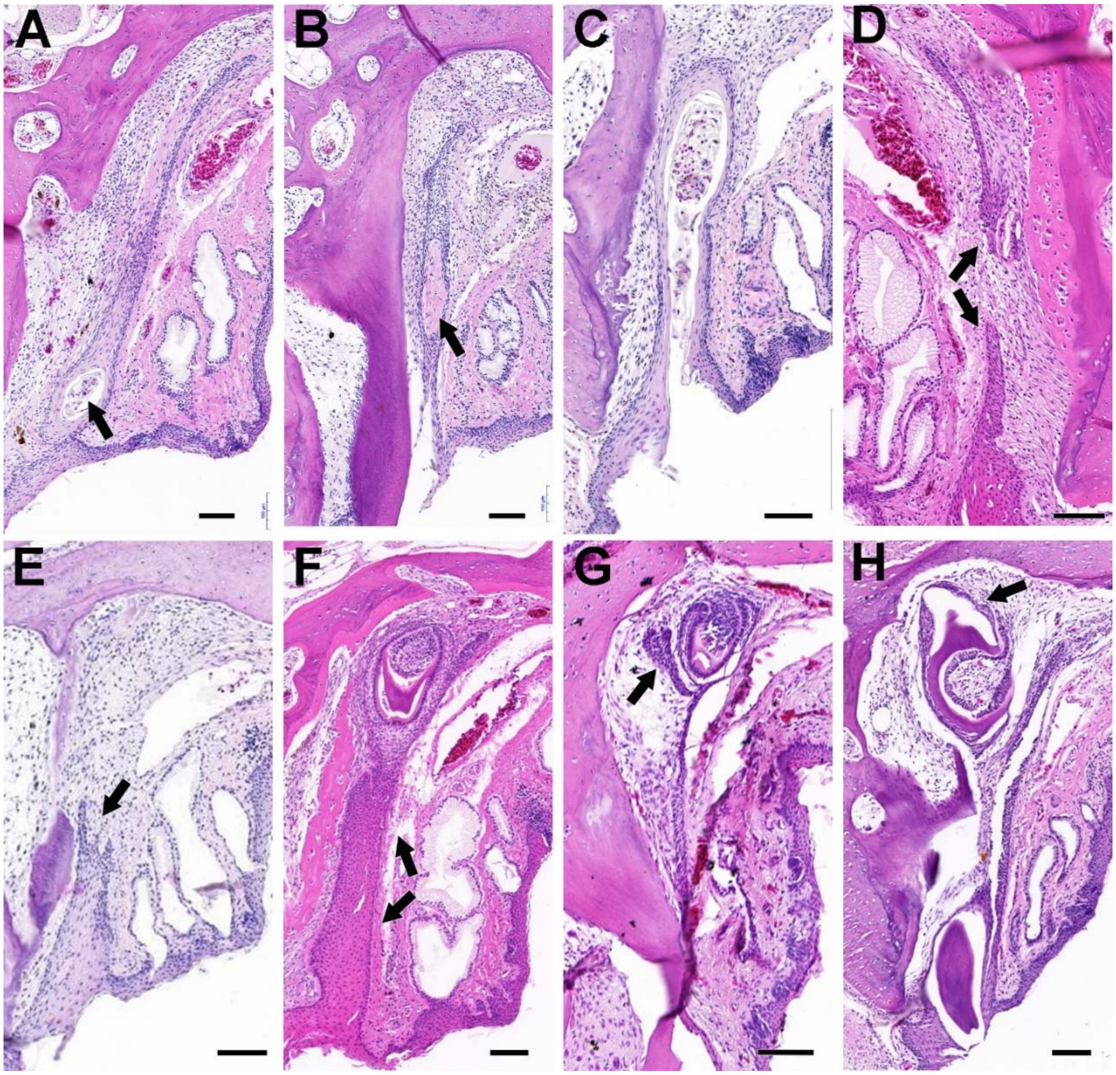

Figure S2: Examples of dental lamina dysmorphology as a result of surgical disruption. A) A cyst in the proximal portion of the dental lamina (arrow), 1 week post-enucleation. B) A cyst in the dental lamina (arrow), 1 week post-enucleation with curettage. C) A large cyst splitting the dental lamina labio-lingually, 1 week post enucleation with curettage. D) The dental lamina is ripped into 2 pieces (arrows), 2 weeks post-enucleation. E) A truncated region of the dental lamina (arrow), 1 week post-enucleation with curettage. F) An area containing a twisted dental lamina that appears thicker than normal (arrows), 2 weeks post-enucleation. G) The successional lamina (arrow) at one tooth position was flipped labially near a developing tooth, 1 month post-enucleation. H) A replacement tooth was flipped upside down, 1 month after curettage (arrow) Corresponding tooth can be seen in the μCT scan rendering in Fig 2D. Scale bars= 100 μm

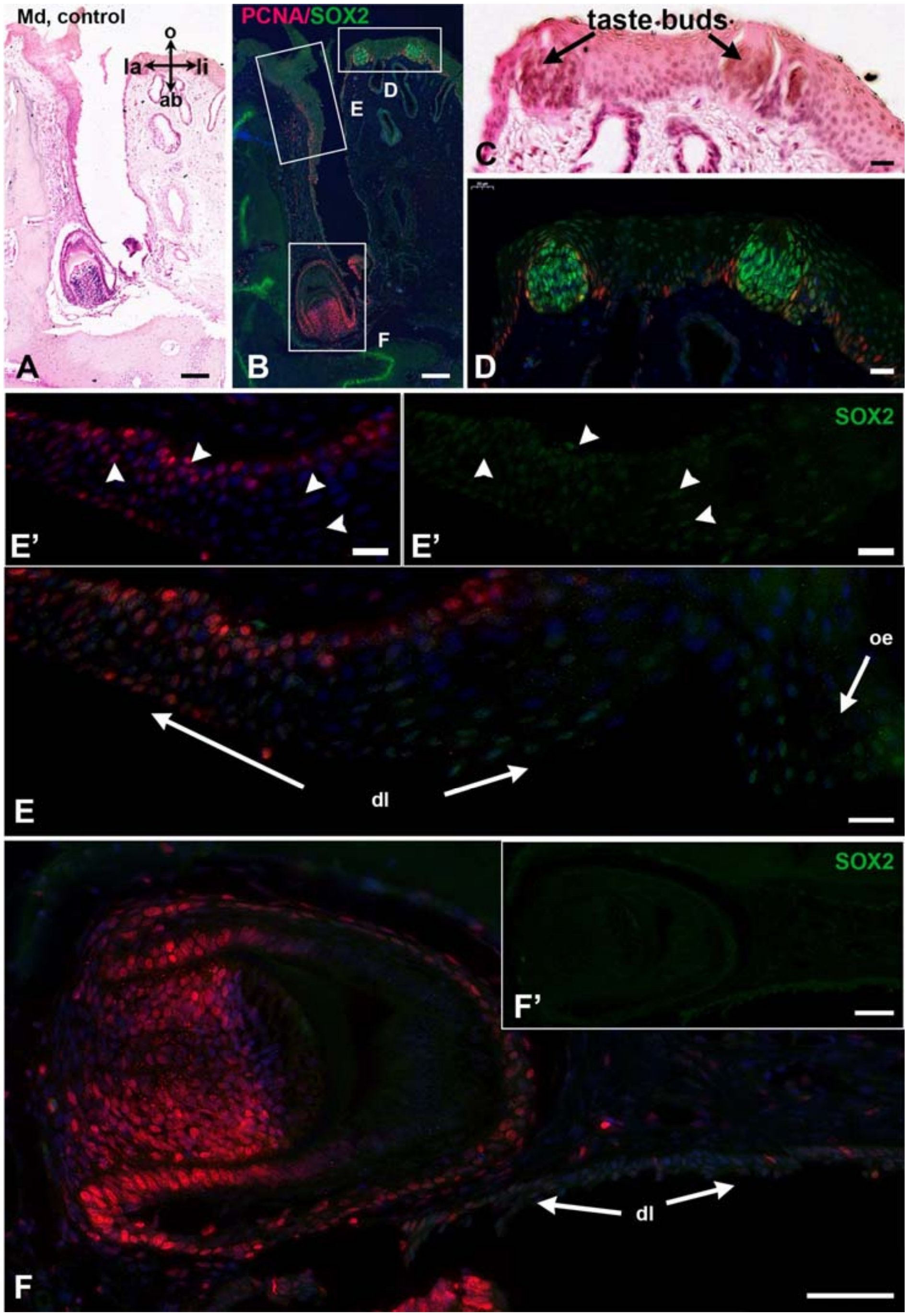

Figure S3: SOX2 expression is expressed weakly in the dental lamina compared to the taste buds. Dual immunostaining with antibodies to SOX2 and PCNA of a control mandibular tooth family. The tear in the tissue next to the dental lamina is an artifact of sectioning. The successional lamina is not included in this section. A,B) A tooth in early bell stage has PCNA positive cells in the enamel organ and dental papilla. C, D) SOX2 and PCNA staining in the taste buds is complementary. E,E') There is very weak SOX2 in nuclei of the dental lamina in comparison to the taste buds. Some of the PCNA signal does overlap SOX2 as seen in the split channels. F,F') PCNA positive cells are present in the dental lamina, cervical loops. No nuclear staining for SOX is present in the enamel organ or adjacent dental lamina (inset). Key: dl, dental lamina. Scale bars for A,B = 100μm, F = 50 μm and C,D, E = 20 μm.

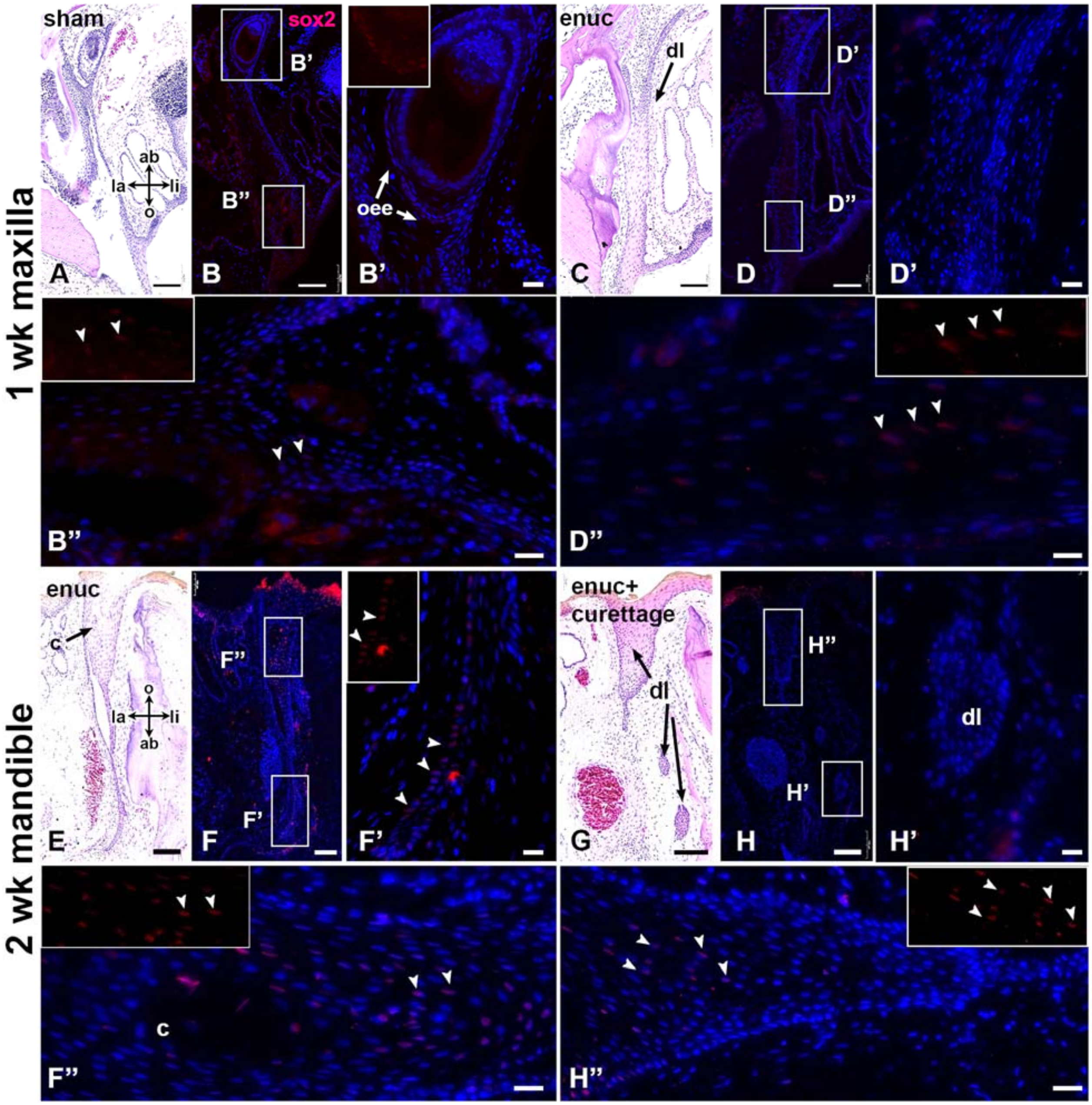

Figure S4: SOX2 antibody staining of dental lamina post-surgery. A-B'') A near-adjacent section stained with H and E. The tooth bud remained in place in the sham surgery. **B-B'')** Weak SOX2 staining is seen the tooth that is in late bell stage (white arrowheads). **C)** Enucleation has removed the 2nd generation tooth. **D-D'')** The dental lamina has very weak SOX2 staining (arrowheads, inset D''). **E)** Two weeks after surgery there is a cyst that has formed in the oral part of the dental lamina. **F-F'')** More noticeable SOX2 staining in some cells of the oral dental lamina (F'', inset). **G)** removal of second generation teeth plus curettage has torn the dental lamina so there are separate islands of epithelium. **H-H'')** SOX2 staining is absent in the aboral remnants of the dental lamina (H'). However, in the oral dental lamina there are a few positive cells (arrowheads, H'', inset). Key: ab, aboral; c, cyst; dl, dental lamina; la, labial; li, lingual; o, oral. Scale bars = 100 microns in A,C,E,G and 20 microns elsewhere.

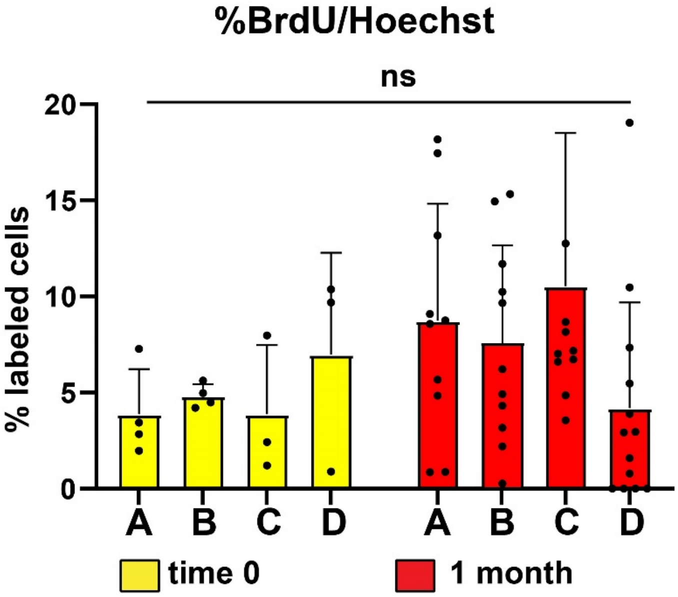

Figure S5: Proportion of cells in the dental lamina labeled with BrdU exclusively. The proportion of BrdU labeled cells is not significantly different between time points and between treatments. Key: A – enucleation, B – enucleation and curettage, C – sham, D – control.

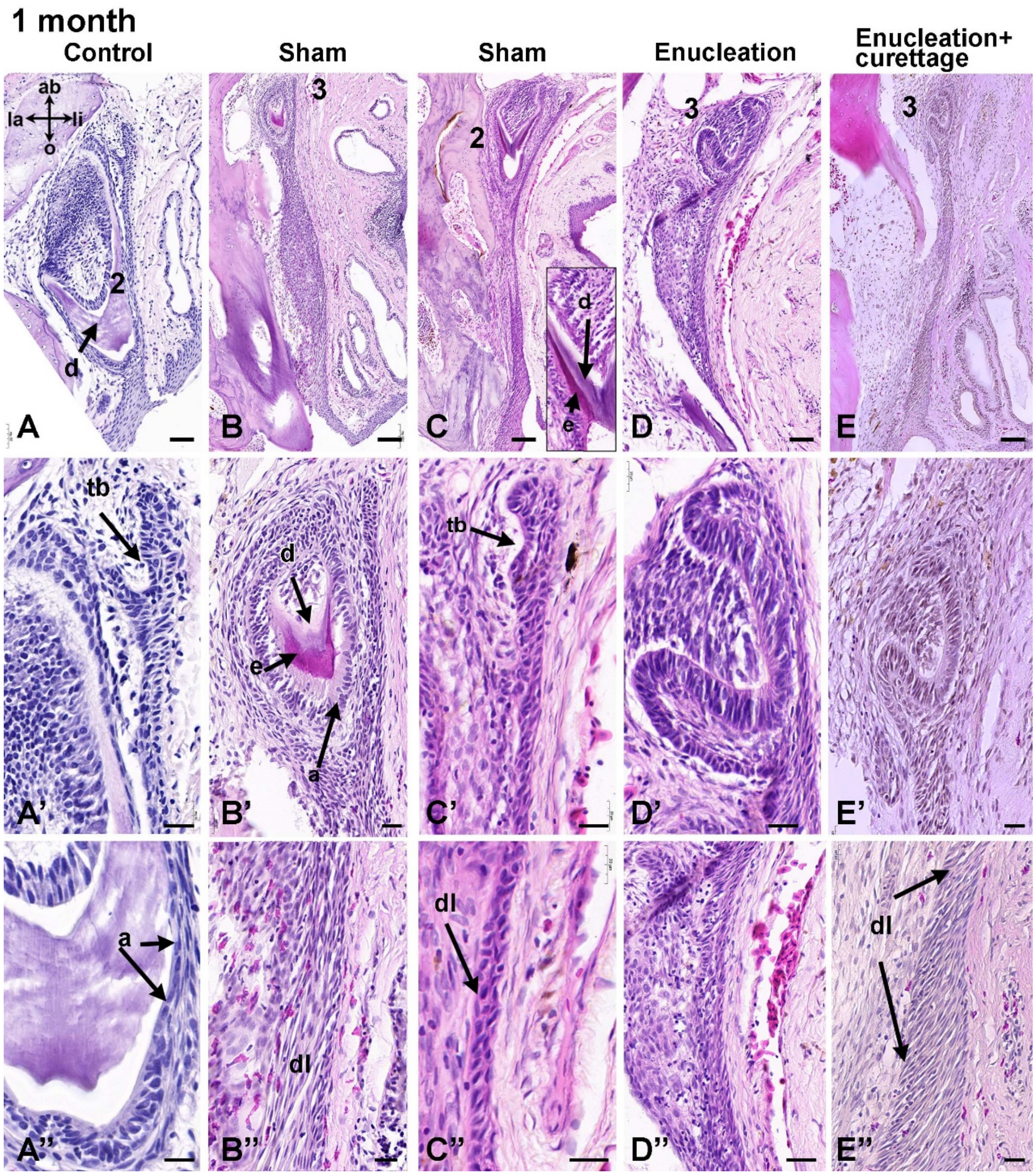

Figure S6: Histology of near-adjacent sections used in Figure 7. A-A'') Control tooth family. The tooth bud formed since the surgery. **B-B'')** Sham tooth that was in bell stage at the time of surgery. A third generation tooth bud has also formed. **C-C'')** Sham teeth, one that was in early bell stage at the time of surgery and a new tooth bud. **D-D'')** Cap stage third generation tooth formed since surgery. **E-E'')** Cap stage third generation tooth formed since the surgery. Key: 2, second generation tooth; 3 , third generation tooth; a , ameloblasts; ab, aboral; d, dentin; dl, dental lamina; e, enamel matrix; la, labial; li, lingual; o, oral; tb, tooth bud. Scale bars = 100 microns in A-E and 20 microns elsewhere.

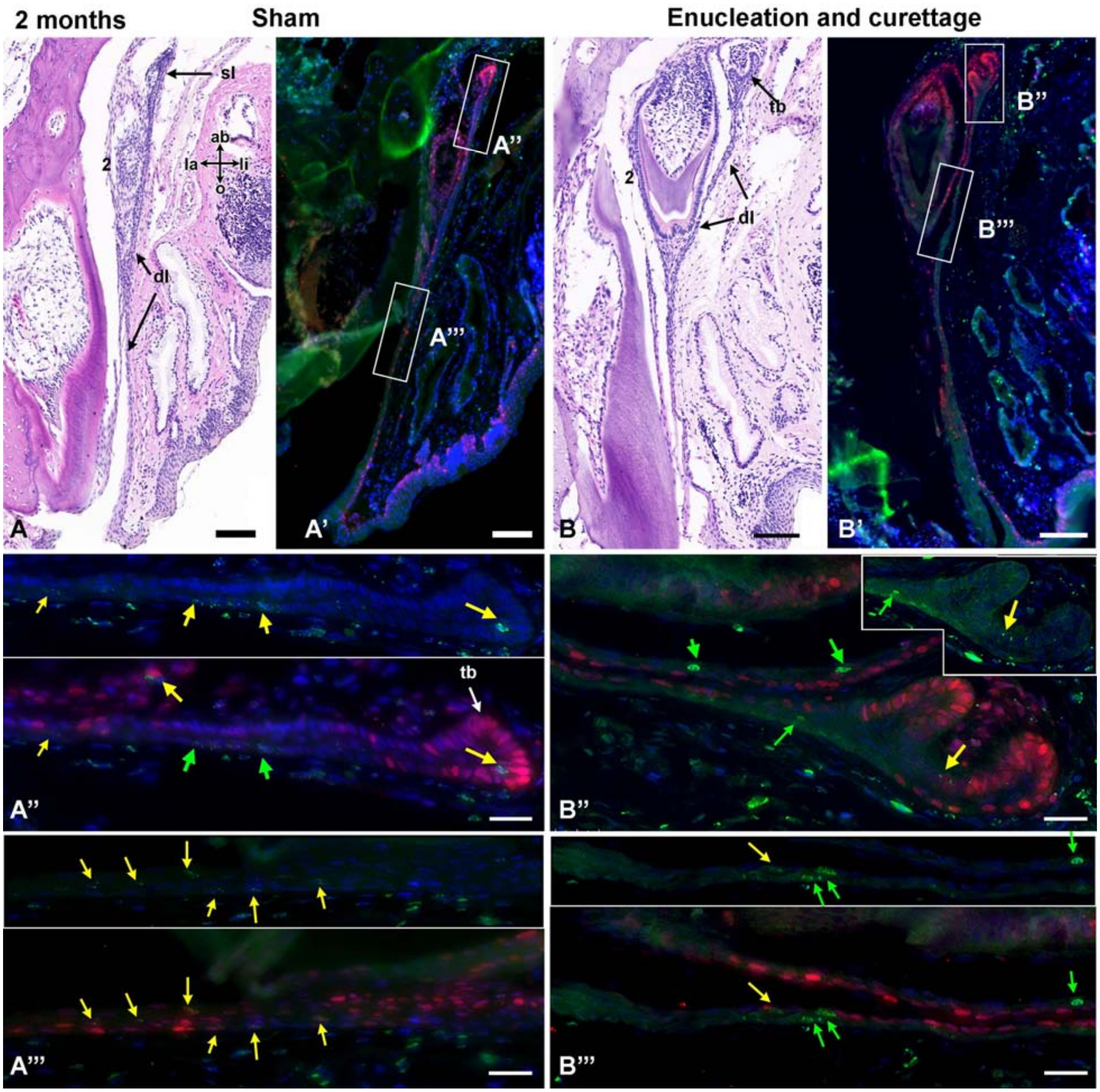

Figure S7: Distribution of BrdU label in bud or cap stage teeth after a 2 month chase. Sections were stained with antibodies to PCNA and BrdU. **A-A''')** A sham treated tooth family with a second generation tooth and a bud stage tooth at the aboral tip of the dental lamina. The PCNA labeling is strongest in the tooth bud epithelium. A'' and inset) The dental lamina adjacent to the tooth bud contains cells with fragmented BrdU. In addition there is one cell in the stellate reticulum that is labeled with BrdU and PCNA. A''') Additional lingual dental lamina cells are labeled with BrdU and PCNA. B-B''') A treated region that has redistributed BrdU in the inner enamel epithelium of the cap stage tooth (B'' plus inset). B''') The dental lamina has retained BrdU cells from the original pulse. Key: 2, second generation tooth; ab, aboral; dl, dental lamina; la, labial; li, lingual; o, oral; tb, tooth bud. Scale bars = 100 microns in A,A'B,B' and 20 microns A'',A''', B'', B'''.

Supplementary Tables

Table S1: Adult gecko tissues used in this study

Code	Sex	Treatment	Jaw	Timepoint	Weight at euthanasia (g)	Experiment
1	F	E, E+C, Sham	max	24 hours	13	CT scan, HE, IF
2	F	E, E+C, Sham	max	24 hours	17	CT scan, HE, IF
3	F	E, E+C, Sham	max	3 days	14	CT scan, HE, IF
4	M	E, E+C, Sham	mand	3 days	74.5	CT scan, HE, IF
5	F	E, E+C, Sham	max	1 week	21	CT scan, HE, IF
6	M	E, E+C, Sham	mand	1 week	32	CT scan, HE
7	F	E, E+C, Sham	mand	1 week	31.5	CT scan, HE
8	M	E, E+C, Sham	mand	1 week	35	CT scan, HE, IF
9	F	E, E+C, Sham	max	1 week	34	Immunostaining, CT scan, Pulse-chase time 0
10	F	E, Sham	max	1 week	44	CT scan, HE, IF
11	F	E, E+C, Sham	max	1 week	59	CT scan, HE, IF
12	F	E, Sham	mand	1 week	41.5	PTA stained CT scan
13	F	E, Sham	mand	1 week	42	CT scan, HE, IF
14	F	E, E+C, Sham	max	2 weeks	61	CT scan, HE, IF
15	F	E, E+C, Sham	mand	2 weeks	31.5	CT scan, HE, IF
16	F	E, E+C, Sham	mand	2 weeks	30.5	CT scan, HE, IF
12	F	E, E+C, Sham	max	2 weeks	41.5	CT scan, HE, IF
13	F	Sham + control	max	2 weeks	42	CT scan, HE, IF
4	M	E, E+C, Sham	max	4 weeks	74.5	CT scan, Pulse-chase 1 month, Xylenol Orange, HE, IF
8	M	E, E+C, Sham	max	4 weeks	35	CT scan, Pulse-chase 1 month, Xylenol Orange, HE, IF
17	F	E, E+C, Sham	max	4 weeks	33.5	CT scan, Pulse-chase 1 month, Xylenol Orange, HE, IF
6	M	E, E+C, Sham	max	8 weeks	32	CT scan, Pulse-chase 2 months, Xylenol Orange, HE, IF
7	F	E, E+C, Sham	max	8 weeks	31.5	CT scan, Pulse-chase 2 months, Xylenol Orange, HE, IF
18	F	E, E+C, Sham	max	8 weeks	33.5	Pulse-chase 2 months, Xylenol Orange, CT scan, HE, IF
15	F	E, E+C, Sham	max	12 weeks	31.5	Xylenol Orange, CT scan, HE
16	F	E, E+C, Sham	max	12 weeks	30.5	Xylenol Orange, CT scan, HE

E= Enucleation, E+C= Enulceation and Curettage, all animals received Calcein 24h prior to treatment, control tissues were taken from all animals

Table S2: Numbers of BrdU and PCNA labeled cells in the dental lamina

animal number	Time point	Treatment replicate #	Hoescht	BrdU+/-PCNA	PCNA	BrdU only	BrdU + PCNA	BrdU+PCNA /PCNA	BrdU/ Hoechst	(BrdU+/-PCNA)/ Hoechst	PCNA/ Hoechst	PCNA+BrdU/ Hoechst
9	1 wk	A1	202	124	175	4	120	68.57%	1.98%	61.39%	86.63%	59.41%
9	1 wk	A2	291	120	168	10	110	65.48%	3.44%	41.24%	57.73%	37.80%
9	1 wk	A3	635	333	472	18	315	66.74%	2.83%	52.44%	74.33%	49.61%
9	1 wk	A4	261	191	232	19	172	74.14%	7.28%	73.18%	88.89%	65.90%
9	1 wk	B1	596	387	431	34	354	82.02%	5.63%	64.99%	72.38%	59.36%
9	1 wk	B2	380	264	331	16	248	74.89%	4.22%	69.43%	87.09%	65.22%
9	1 wk	B3	468	272	300	21	251	83.67%	4.49%	58.12%	64.10%	53.63%
9	1 wk	B4	422	212	271	21	191	70.48%	4.98%	50.24%	64.22%	45.26%
9	1 wk	C1	250	127	213	3	124	58.22%	1.20%	50.80%	85.20%	49.60%
9	1 wk	C2	213	109	176	17	92	52.27%	7.98%	51.17%	82.63%	43.19%
9	1 wk	C3	247	91	155	6	85	54.84%	2.43%	36.84%	62.75%	34.41%
9	1 wk	D1	446	86	226	4	82	36.28%	0.90%	19.28%	50.67%	18.39%
9	1 wk	D2	289	41	57	28	13	22.81%	9.69%	14.19%	19.72%	4.50%
9	1 wk	D3	270	60	103	28	32	31.07%	10.37%	22.22%	38.15%	11.85%
5	1 wk	A5	274	NA	151	NA	NA	NA	NA	NA	55.11%	NA
5	1 wk	A6	721	NA	462	NA	NA	NA	NA	NA	64.08%	NA
5	1 wk	A7	860	NA	345	NA	NA	NA	NA	NA	40.12%	NA
5	1 wk	A8	381	NA	108	NA	NA	NA	NA	NA	28.35%	NA
5	1 wk	B5	317	NA	130	NA	NA	NA	NA	NA	41.01%	NA
5	1 wk	B6	545	NA	246	NA	NA	NA	NA	NA	45.14%	NA
5	1 wk	B7	430	NA	219	NA	NA	NA	NA	NA	50.93%	NA
5	1 wk	B8	262	NA	192	NA	NA	NA	NA	NA	73.28%	NA
5	1 wk	C4	349	NA	143	NA	NA	NA	NA	NA	40.97%	NA
5	1 wk	C5	170	NA	113	NA	NA	NA	NA	NA	66.47%	NA
5	1 wk	C6	319	NA	207	NA	NA	NA	NA	NA	64.89%	NA
5	1 wk	C7	323	NA	204	NA	NA	NA	NA	NA	63.16%	NA
5	1 wk	D4	639	NA	458	NA	NA	NA	NA	NA	71.67%	NA
5	1 wk	D5	475	NA	306	NA	NA	NA	NA	NA	64.42%	NA
5	1 wk	D6	154	NA	85	NA	NA	NA	NA	NA	55.19%	NA
5	1 wk	D7	706	NA	467	NA	NA	NA	NA	NA	66.15%	NA
11	1 wk	A9	438	NA	230	NA	NA	NA	NA	NA	52.51%	NA
11	1 wk	A10	317	NA	157	NA	NA	NA	NA	NA	49.53%	NA
11	1 wk	A11	579	NA	322	NA	NA	NA	NA	NA	55.61%	NA
11	1 wk	A12	703	NA	316	NA	NA	NA	NA	NA	44.95%	NA
11	1 wk	B9	731	NA	350	NA	NA	NA	NA	NA	47.88%	NA
11	1 wk	B10	592	NA	323	NA	NA	NA	NA	NA	54.56%	NA
11	1 wk	B11	305	NA	173	NA	NA	NA	NA	NA	56.72%	NA
11	1 wk	B12	303	NA	166	NA	NA	NA	NA	NA	54.79%	NA
11	1 wk	C8	563	NA	308	NA	NA	NA	NA	NA	54.71%	NA
11	1 wk	C9	335	NA	122	NA	NA	NA	NA	NA	36.42%	NA
11	1 wk	C10	230	NA	109	NA	NA	NA	NA	NA	47.39%	NA
11	1 wk	C11	302	NA	194	NA	NA	NA	NA	NA	64.24%	NA
11	1 wk	D8	319	NA	146	NA	NA	NA	NA	NA	45.77%	NA
11	1 wk	D9	375	NA	210	NA	NA	NA	NA	NA	56.00%	NA
11	1 wk	D10	137	NA	65	NA	NA	NA	NA	NA	47.45%	NA
4	1 month	A1	702	66	173	10	57	32.82%	1.38%	9.44%	24.58%	8.07%
4	1 month	A2	684	43	245	6	37	15.10%	0.88%	6.29%	35.82%	5.41%
4	1 month	A3	423	41	233	24	17	7.30%	5.67%	9.69%	55.08%	4.02%
4	1 month	B1	495	57	264	24	33	12.50%	4.85%	11.52%	53.33%	6.67%
4	1 month	B2	658	33	227	14	19	8.37%	2.13%	5.02%	34.50%	2.89%
4	1 month	B3	940	137	426	29	108	25.35%	3.09%	14.57%	45.32%	11.49%
4	1 month	C1	357	37	186	24	13	6.99%	6.72%	10.36%	52.10%	3.64%
4	1 month	C2	185	12	39	9	3	7.69%	4.86%	6.49%	21.08%	1.62%

A - enucleation, B - enucleation+curettage, C - sham, D - control

Table S2: Numbers of BrdU and PCNA labeled cells in the dental lamina

animal number	Time point	Treatment replicate #	Hoescht	BrdU+/-PCNA	PCNA	BrdU only	BrdU + PCNA	BrdU+PCNA /PCNA	BrdU/ Hoechst	(BrdU+/-PCNA)/ Hoechst	PCNA/ Hoechst	PCNA+BrdU/ Hoechst
4	1 month	C3	232	24	55	16	8	13.86%	7.03%	10.33%	23.82%	3.30%
4	1 month	D1	300	0	106	0	0	0.00%	0.00%	0.00%	35.33%	0.00%
4	1 month	D2	399	0	119	0	0	0.00%	0.00%	0.00%	29.82%	0.00%
4	1 month	D3	450	0	101	0	0	0.00%	0.00%	0.00%	22.44%	0.00%
17	1 month	A4	659	163	345	115	48	13.91%	17.45%	24.73%	52.35%	7.28%
17	1 month	A5	407	107	160	74	33	20.63%	18.18%	26.29%	39.31%	8.11%
17	1 month	A6	372	62	145	49	13	8.97%	13.17%	16.67%	38.98%	3.49%
17	1 month	B4	400	104	194	60	44	10.57%	15.00%	26.00%	48.50%	11.00%
17	1 month	B5	216	34	123	21	13	10.57%	9.72%	15.74%	56.94%	6.02%
17	1 month	B6	221	38	72	26	13	18.06%	11.76%	17.19%	32.58%	5.88%
17	1 month	C4	56	21	26	17	4	15.38%	30.36%	37.50%	46.43%	7.14%
17	1 month	C5	150	28	50	13	15	30.00%	8.67%	18.67%	33.33%	10.00%
17	1 month	C6	400	74	195	51	23	11.79%	12.75%	18.50%	48.75%	5.75%
17	1 month	C7	149	46	90	30	16	17.78%	20.13%	30.87%	60.40%	10.74%
17	1 month	D4	147	36	60	28	8	13.33%	19.05%	24.49%	40.82%	5.44%
17	1 month	D5	271	14	124	8	6	4.84%	2.95%	5.17%	45.76%	2.21%
17	1 month	D6	458	84	243	48	36	14.81%	10.48%	18.34%	53.06%	7.86%
17	1 month	D7	548	25	190	16	9	4.74%	2.92%	4.56%	34.67%	1.64%
17	1 month	D8	347	25	122	19	6	4.92%	5.48%	7.20%	35.16%	1.73%
17	1 month	D9	231	16	138	9	7	5.07%	3.90%	6.93%	59.74%	3.03%
17	1 month	D10	177	17	60	13	4	6.67%	7.34%	9.60%	33.90%	2.26%
8	1 month	A7	245	21	79	21	0	0.00%	8.57%	8.57%	32.24%	0.00%
8	1 month	A8	231	20	75	2	18	24.00%	0.87%	8.66%	32.47%	7.79%
8	1 month	A9	264	24	56	24	0	0.00%	9.09%	9.09%	21.21%	0.00%
8	1 month	A10	320	30	76	28	2	2.63%	8.75%	9.38%	23.75%	0.63%
8	1 month	B6	526	22	250	1	21	8.40%	0.19%	4.18%	47.53%	3.99%
8	1 month	B7	330	18	80	14	4	5.00%	4.24%	5.45%	24.24%	1.21%
8	1 month	B8	117	19	32	18	1	3.13%	15.38%	16.24%	27.35%	0.85%
8	1 month	B9	309	22	76	19	3	3.95%	6.15%	7.12%	24.60%	0.97%
8	1 month	B10	165	18	52	17	1	1.92%	10.30%	10.91%	31.52%	0.61%
8	1 month	C8	279	23	72	20	3	4.17%	7.17%	8.24%	25.81%	1.08%
8	1 month	C9	233	22	63	19	3	4.76%	8.15%	9.44%	27.04%	1.29%
8	1 month	C10	324	22	126	12	10	7.97%	3.55%	6.65%	38.79%	3.09%
8	1 month	C11	378	26	69	25	1	1.45%	6.61%	6.88%	18.25%	0.26%
8	1 month	D11	189	13	110	3	10	9.09%	1.59%	6.88%	58.20%	5.29%
8	1 month	D12	126	9	63	1	8	12.70%	0.79%	7.14%	50.00%	6.35%
8	1 month	D13	75	2	34	0	2	5.88%	0.00%	2.67%	45.33%	2.67%

A - enucleation, B - enucleation+curettage, C - sham, D - control

Table S3: Proliferation in the dental lamina. Adjusted P-values from 2-way ANOVA with Tukey's post-hoc test of all pair-wise comparisons

A) (BrdU+/-PCNA)/Hoechst

	Time 0				1 month			
% label±1 SD	**A_0 57.1±13.5**	**B_0 60.7±8.4**	**C_0 46.3±8.2**	**D_0 18.6±4.1**	**A_1 13.1±7.1**	**B_1 12.2±6.7**	**C_1 14.9±10.5**	**D_1 7.2±7.2**
**A_0**		0.9984	0.6909	<0.0001	<0.0001	<0.0001	<0.0001	<0.0001
**B_0**	0.9984		0.3286	<0.0001	<0.0001	<0.0001	<0.0001	<0.0001
**C_0**	0.6909	0.3286		0.0038	<0.0001	<0.0001	<0.0001	<0.0001
**D_0**	<0.0001	<0.0001	0.0038		0.9738	0.9351	0.9975	0.4064
**A_1**	<0.0001	<0.0001	<0.0001	0.9738		>0.9999	0.9996	0.6848
**B_1**	<0.0001	<0.0001	<0.0001	0.9351	>0.9999		0.9938	0.8189
**C_1**	<0.0001	<0.0001	<0.0001	0.9975	0.9996	0.9938		0.3262
**D_1**	<0.0001	<0.0001	<0.0001	0.4064	0.6848	0.8189	0.3262	

B) PCNA/Hoechst

	Time 0				1 month			
% label ±1 SD	**A_0 58.2±17.9**	**B_0 59.4±13.6**	**C_0 60.8±15.3**	**D_0 51.5±15.1**	**A_1 35.7±11.3**	**B_1 38.8±11.9**	**C_1 35.7±14.4**	**D_1 41.9±11.3**
**A_0**		>0.9999	0.9998	0.9542	0.0079	0.0289	0.0057	0.0861
**B_0**	>0.9999		>0.9999	0.8900	0.0040	0.0152	0.0028	0.0479
**C_0**	0.9998	>0.9999		0.7979	0.0024	0.0094	0.0017	0.0302
**D_0**	0.9542	0.8900	0.7979		0.2021	0.4365	0.1774	0.7276
**A_1**	0.0079	0.0040	0.0024	0.2021		0.9996	>0.9999	0.9662
**B_1**	0.0289	0.0152	0.0094	0.4365	0.9996		0.9996	0.9994
**C_1**	0.0057	0.0028	0.0017	0.1774	>0.9999	0.9996		0.9602
**D_1**	0.0861	0.0479	0.0302	0.7276	0.9662	0.9994	0.9602	

C) BrdU+PCNA/Hoechst

	Time 0				1 month			
% label±1 SD	**A_0 53.2±12.2**	**B_0 55.9±8.6**	**C_0 42.4±7.6**	**D_0 11.6±6.9**	**A_1 4.7±3.7**	**B_1 4.7±3.9**	**C_1 4.4±3.6**	**D_1 2.9±2.5**
**A_0**		0.9948	0.1387	<0.0001	<0.0001	<0.0001	<0.0001	<0.0001
**B_0**	0.9948		0.0253	<0.0001	<0.0001	<0.0001	<0.0001	<0.0001
**C_0**	0.1387	0.0253		<0.0001	<0.0001	<0.0001	<0.0001	<0.0001
**D_0**	<0.0001	<0.0001	<0.0001		0.4842	0.4648	0.4087	0.1805
**A_1**	<0.0001	<0.0001	<0.0001	0.4842		>0.9999	>0.9999	0.9920
**B_1**	<0.0001	<0.0001	<0.0001	0.4648	>0.9999		>0.9999	0.9915
**C_1**	<0.0001	<0.0001	<0.0001	0.4087	>0.9999	>0.9999		0.9974
**D_1**	<0.0001	<0.0001	<0.0001	0.1805	0.9920	0.9915	0.9974	

D) BrdU+PCNA/PCNA

	Time 0				1 month			
% label±1 SD	**A_0 68.7±3.8**	**B_0 77.9±6.2**	**C_0 55.1±2.9**	**D_0 30.1±6.8**	**A_1 13.2±12.5**	**B_1 10.9±7.9**	**C_1 12.3±9.7**	**D_1 6.3±4.9**
**A_0**		0.7741	0.4028	<0.0001	<0.0001	<0.0001	<0.0001	<0.0001
**B_0**	0.7741		0.0161	<0.0001	<0.0001	<0.0001	<0.0001	<0.0001
**C_0**	0.4028	0.0161		0.0120	<0.0001	<0.0001	<0.0001	<0.0001
**D_0**	<0.0001	<0.0001	0.0120		0.0626	0.0186	0.0373	0.0011
**A_1**	<0.0001	<0.0001	<0.0001	0.0626		0.9981	>0.9999	0.5074
**B_1**	<0.0001	<0.0001	<0.0001	0.0186	0.9981		>0.9999	0.8765
**C_1**	<0.0001	<0.0001	<0.0001	0.0373	>0.9999	>0.9999		0.6512
**D_1**	<0.0001	<0.0001	<0.0001	0.0011	0.5074	0.8765	0.6512	

Key: +/- with or without, A – enucleation, B – enucleation plus curettage, C – sham, D – control

Table S4: Numbers of BrdU label-retaining and PCNA positive cells in different regions of the dentition after a 2-month chase

		DL		IEE	OEE	SR
**Treatment Replicate #**	**BrdU only**	**BrdU+PCNA**	**BrdU+/- PCNA**	**BrdU+/- PCNA**	**BrdU+/- PCNA**	**BrdU+/- PCNA**
A1	12	26	38	7	2	5
A2	9	62	71	NA	NA	NA
A3	10	35	45	0	4	NA
B1	16	28	44	13	5	3
B2	6	14	20	5	8	1
B3	3	7	10	0	0	0
B4	6	1	7	0	0	0
C1	0	4	4	1	2	0
C2	1	8	9	0	0	1
C3	1	5	6	5	6	1
C4	12	49	61	NA	NA	NA
C5	9	6	15	NA	NA	NA
D1	14	6	20	3	0	0
D2	10	6	16	1	3	3
D3	8	6	14	0	0	1

Key: +/- with or without PCNA label, A – enucleation, B – enucleation plus curettage, C – sham, D – control, DL – dental lamina, IEE – inner enamel epithelium, OEE – outer enamel epithelium, NA – no tooth bud in section, SR – stellate reticulum
